# Structure‐Activity Relationships of Benzamides and Isoindolines Designed as SARS‐CoV Protease Inhibitors Effective against SARS‐CoV‐2

**DOI:** 10.1002/cmdc.202000548

**Published:** 2020-10-16

**Authors:** Armin Welker, Christian Kersten, Christin Müller, Ramakanth Madhugiri, Collin Zimmer, Patrick Müller, Robert Zimmermann, Stefan Hammerschmidt, Hannah Maus, John Ziebuhr, Christoph Sotriffer, Tanja Schirmeister

**Affiliations:** ^1^ Institute for Pharmacy and Food Chemistry Justus Maximilians University Würzburg Am Hubland 97074 Würzburg Germany; ^2^ Institute for Pharmaceutical and Biomedical Sciences Johannes Gutenberg University Mainz Staudingerweg 5 55128 Mainz Germany; ^3^ Institute of Medical Virology Justus Liebig University Giessen Schubertstrasse 81 35392 Giessen Germany

**Keywords:** Antiviral agents, Computational chemistry, Drug design, Protease inhibitors, Structure-activity relationships

## Abstract

Inhibition of coronavirus (CoV)‐encoded papain‐like cysteine proteases (PL^pro^) represents an attractive strategy to treat infections by these important human pathogens. Herein we report on structure‐activity relationships (SAR) of the noncovalent active‐site directed inhibitor (*R*)‐5‐amino‐2‐methyl‐N‐(1‐(naphthalen‐1‐yl)ethyl) benzamide (**2 b**), which is known to bind into the S3 and S4 pockets of the SARS‐CoV PL^pro^. Moreover, we report the discovery of isoindolines as a new class of potent PL^pro^ inhibitors. The studies also provide a deeper understanding of the binding modes of this inhibitor class. Importantly, the inhibitors were also confirmed to inhibit SARS‐CoV‐2 replication in cell culture suggesting that, due to the high structural similarities of the target proteases, inhibitors identified against SARS‐CoV PL^pro^ are valuable starting points for the development of new pan‐coronaviral inhibitors.

## Graphical abstract

**Valuable starting points**: Within this study, the effectivity of naphthalene‐based inhibitors, formerly developed against the papain‐like protease (PL^pro^) of the severe acute respiratory syndrome (SARS)‐causing coronavirus (CoV), against the homologous PL^pro^ of SARS‐CoV‐2 is shown. Furthermore, isoindoline is presented as an innovative scaffold for this inhibitor type. In particular, high antiviral activity combined with low cytotoxicity are highlights of these inhibitors.

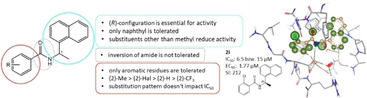

## Introduction

Within the past two decades, members of the genus *Betacoronavirus* have caused three major outbreaks of severe respiratory disease in humans, including SARS (severe acute respiratory syndrome), MERS (Middle East respiratory syndrome), and coronavirus disease 19 (COVID‐19).[^1^, ^2^, ^3^, ^4^, ^5^, ^6^, ^7^] There is strong evidence that all these newly emerging coronaviruses have their natural reservoir in animals. Given the remarkable zoonotic potential of these viruses and the major impact of infections caused by these viruses on human health and the global economy, there is an urgent need to develop broadly acting antivirals that, ideally, should be effective against previously known and newly emerging coronaviruses. The viruses responsible for the SARS outbreak in 2002/2003 and the ongoing COVID‐19 pandemic are called SARS coronavirus (SARS‐CoV) and SARS coronavirus 2 (SARS‐CoV‐2), respectively. Genetically, the two viruses are very closely related and are members of the same coronavirus species called *Severe acute respiratory syndrome‐related coronavirus* (subgenus *Sarbecovirus*, genus *Betacoronavirus*).^[8]^

To date, no causative treatment options for SARS, COVID‐19 and MERS^[9]^ have been approved.^[10]^ Therefore, an improved understanding of structure‐activity relationships (SAR) of inhibitors targeting critical steps in viral replication is of utmost importance and expected to facilitate drug development. In this context, viral proteases have been identified as promising targets for inhibiting the replication of viruses of diverse families, such as *Coronaviridae*, *Flaviviridae*, *Retroviridae*, and *Picornaviridae*. Inhibitors of viral proteases are highly effective drugs and are widely used in clinical practice, for example, in the treatment of HIV/AIDS and hepatitis C infections. Like many other positive‐strand RNA viruses, CoVs express large polyproteins (called pp1a and pp1ab) that are processed by viral proteases at multiple sites.^[11]^ In SARS‐like CoVs, pp1a and pp1ab are processed by two proteases, namely the coronavirus main protease (M^pro^, also called 3 C‐like protease, 3CL^pro^) and papain‐like protease (PL^pro^). Upon cleavage, they release a total of 16 nonstructural proteins (nsp 1 to 16) from pp1a and pp1ab.^[12]^ The vast majority if these nsps is involved in the formation of membrane‐anchored multi‐subunit protein complexes that are referred to as coronavirus replication‐transcription complexes (RTCs) which replicate the viral genome RNA and produce an extensive set of subgenomic mRNAs, the latter encoding viral structural and several accessory proteins.[^11^, ^13^] Due to their key role in the production of active RTCs, coronavirus proteases are considered as promising drug targets.[^14^, ^15^]

**Scheme 1:**
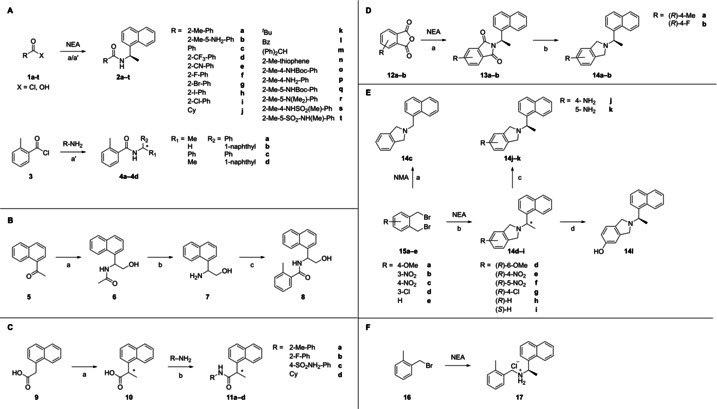
Compounds used for SAR. (**A**): Synthesis of benzamides **2 a**–**t**. Reagents and conditions: X=OH a) appropriate solvent, HOBt‐hydrate, coupling reagent, (*R*)‐naphthylethylamine (NEA). X=Cl a’) NEA, Et_2_O/H_2_O, Na_2_CO_3_, r.t. B: Synthesis of **8**. (**B**): Reagents and conditions: a) 1. benzene, *N*‐bromosuccinimide, *p*‐toluoenesulfonic acid monohydrate, reflux, 16 h. 2. H_2_O, sodium acetate trihydrate, ethanol, reflux, 2 h. 3. isopropanol, ammonia acetate, sodium cyanoborohydride, 95 °C, 1 h. b) 1. semi‐concentrated hydrochloric acid, reflux, 2 d. 2. ethyl acetate, TEA, 2‐methyl benzoyl chloride, r.t., 1 d. c) lithium hydroxide monohydrate, THF/H2O (3 : 1), reflux, 2 d. (**C**): Synthesis of compounds with inverted amide bond. Reagents and conditions: a) 1. MeOH, H_2_SO_4_, r.t.‐65 °C, 2 h. 2. MeI, DMSO, KOH, r.t., 10 d. 3. LiOH, THF, H_2_O, r.t., 16 h. b) HOBt‐hydrate, DCC, 0 °C‐r.t., 1 d. (**D**): Synthesis of isoindoline derivatives **14 a**–**b**. Reagents and conditions: a) NEA, heating with a heat gun. b) THF abs., LAH, r.t.‐70 °C, 2 h. (**E**): Synthesis of isoindoline derivatives **14 c**–**l**. Reagents and conditions: a) Naphthylmethylamine (NMA), EtOAc, Na_2_CO_3_, r.t.‐77 °C, 1 h. b) NEA, toluene, TEA, 120 °C, 1 h. c) SnCl_2_ ⋅ 2H_2_O, HCl, r.t.‐100 °C, 2 h. d) HBr, acetic acid, 120 °C, 24 h. (**F**) Synthesis of the acyclic secondary amine derivative. Reagents and conditions: Toluene, K_2_CO_3_, 110 °C, 12 h.

PL^pro^ is a cysteine protease with a classical Cys‐His‐Asp catalytic triad (Cys112, His273, Asp287) and a precatalytically deprotonated thiolate that acts as the nucleophile for proteolysis.^[16]^ The protease cleaves three sites with a LXGG↓ signature sequence in the N‐proximal regions of pp1a and pp1ab (namely nsp1↓nsp2↓nsp3↓nsp4). In accordance with recent reports,[^17^, ^18^] our sequence and structural alignments revealed that the SARS‐CoV‐2 PL^pro^ shares a high sequence identity and similar fold with its SARS‐CoV homolog (sequence identity 82 %, similarity 90 %, C_α‐_RMSD 1.8 Å, SARS‐CoV PL^pro^ PDB‐ID 3E9S, SARS‐CoV‐2 PL^pro^ PDB‐ID 6WUU^[19]^), especially in the binding site of the inhibitors reported herein, where only two amino acids differ. Those different amino acids, T275/K274 and V301/I300 (labeled as SARS‐CoV/SARS‐CoV‐2 PL^pro^) orient their side‐chains away from the binding site not influencing direct protein‐ligand interactions. Herein we report SARs for a series of rationally designed competitive, noncovalent, nonpeptidic active site‐directed SARS‐CoV PL^pro^ inhibitors which, most likely, also apply to SARS‐CoV‐2 PL^pro^, as supported by virus inhibition data in cell culture and IC_50_ values of a representative set of inhibitors on isolated SARS‐CoV‐2 PL^pro^ in an enzyme‐activity assay. Thus, the information obtained in this study may form the basis for designing pan‐(beta)coronavirus‐PL^pro^ inhibitors. Scheme 1 gives an overview of synthetic routes for the synthesis of inhibitors used for our SAR studies.

## Results and Discussion

### SAR studies with SARS‐CoV PL^pro^

The compounds were assayed to deduce SAR, thereby extending the current knowledge about PL^pro^‐inhibitor interactions. To validate our findings, the previously published IC_50_ values^[20]^ of already known inhibitors **2 a** and **2 b** were compared to the IC_50_ values determined in our laboratory. The data are in good agreement (**2 a**: 3.3 vs. 2.3 μM; **2 b**: 1.1 vs. 0.6 μM) proving the validity of our enzymatic assay. The results of the first assayed set of new compounds (Table 1) indicated that (*R*)‐configuration on the chiral center is crucial for binding affinity (compare **2 a** with its enantiomer **4 d**). Substituents other than a methyl group^[21]^ in position R^1^ are not well tolerated since compounds **4 b** and **4 c**, in which the methyl group was substituted with hydrogen or phenyl, were inactive. This is further confirmed by the inactivity of compound **8**, harboring a hydroxymethyl substituent. Furthermore, an altered substitution position of the naphthalene moiety (**18** and **19**) and substitution of the naphthalene residue with phenyl (compound **4 a**) also resulted in a loss of activity.

**Table 1:** Inhibition of SARS‐CoV PL^pro^ by (benz)amides and anilides.

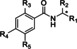								
Compd	R_1_	R_2_	R_3_	R_4_	R_5_	Configuration	IC_50_ [μM]/%^[a]^	
							SARS‐CoV PL^pro^	SARS‐CoV‐2 PL^pro^
**2 a**	CH_3_	1‐naphthyl	CH_3_	H	H	(*R*)	2.3±0.1^[20]^	6.9±0.7
**2 b**	CH_3_	1‐naphthyl	CH_3_	H	NH_2_	(*R*)	0.6±0.1^[20]^	
**18**	CH_3_	2‐naphthyl	CH_3_	H	H	(*S*)	8.7±0.7^[20]^	
**19**	CH_3_	2‐naphthyl	C_2_H_5_	H	H	rac.	>200^[20]^	
**4 a**	CH_3_	phenyl	CH_3_	H	H	rac.	n.a.^[b]^	
**4 b**	H	1‐naphthyl	CH_3_	H	H	‐	n.a.^[b]^	
**4 c^[c]^**	phenyl	phenyl	CH_3_	H	H	‐	n.a.^[b]^	
**4 d**	CH_3_	1‐naphthyl	CH_3_	H	H	(*S*)	n.a.^[b]^	n.a.^[b]^
**8**	CH_2_OH	1‐naphthyl	CH_3_	H	H	rac.	n.a.^[b]^	
**2 c**	CH_3_	1‐naphthyl	H	H	H	(*R*)	62±5	60 %
**2 d**	CH_3_	1‐naphthyl	CF_3_	H	H	(*R*)	n.a.^[b]^	
**2 e**	CH_3_	1‐naphthyl	CN	H	H	(*R*)	18±2	n.a.^[b]^
**2 f**	CH_3_	1‐naphthyl	F	H	H	(*R*)	9±1	67 %
**2 g**	CH_3_	1‐naphthyl	Br	H	H	(*R*)	11±2	73 %
**2 h^[c]^**	CH_3_	1‐naphthyl	I	H	H	(*R*)	25±3	n.a.^[b]^
**2 i**	CH_3_	1‐naphthyl	Cl	H	H	(*R*)	6.5±0.8	15±2
**2 j**	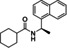					(*R*)	n.a.^[b]^	
**2 k**	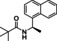					(*R*)	n.a.^[b]^	
**2 l**	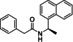					(*R*)	57 %	
**2 m**	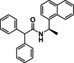					(*R*)	n.a.^[b]^	
**2 n**	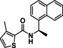					(*R*)	14±1	42±6
**2 o**	CH_3_	1‐naphthyl	CH_3_	NH‐Boc	H	(*R*)	1.6±0.3	
**2 p**	CH_3_	1‐naphthyl	CH_3_	NH_2_	H	(*R*)	1.2+0.1	7.5±0.6
**2 q**	CH_3_	1‐naphthyl	CH_3_	H	NH‐Boc	(*R*)	4.6±0.4	
**2 r^[c]^**	CH_3_	1‐naphthyl	CH_3_	H	N(CH_3_)_2_	(*R*)	4.0±0.2	10±2
**2 s**	CH_3_	1‐naphthyl	CH_3_	NH‐SO_2_‐CH_3_	H	(*R*)	4.8±0.3	10±1
**2 t^[c]^**	CH_3_	1‐naphthyl	CH_3_	H	SO_2_‐NH‐CH_3_	(*R*)	4.0±0.3	87 %
**11 a**	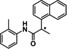					rac.	n.a.^[b]^	n.a.^[b]^
**11 b**	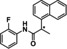					rac.	n.a.^[b]^	n.a.^[b]^
**11 c**	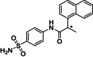					rac.	n.a.^[b]^	n.a.^[b]^
**11 d** ^[c]^	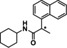					rac.	n.a.^[b]^	n.a.^[b]^

[a] The assays were performed in duplicate. Data are reported as mean±standard deviation. Percentage inhibition was measured at 100 μM inhibitor concentration. [b] Compounds with inhibitory effect less than 50 % at 100 μM compared to DMSO were labelled as not active (n.a.). [c] Compounds could only be obtained with a purity of 90–95 %. To complete the SAR study, the inhibitory effect was nevertheless tested.

In another set of compounds, the effect of *ortho* substituents of the benzamide moiety was investigated (Table 1, **2 c**–**i**). Here, we found that substituents at that position are generally tolerated and even preferred if they follow some steric and electronic characteristics. In contrast to what was expected from the comparison of the compounds **18** and **19**, in which the larger ethyl substituent resulted in a loss of activity,^[20]^ the IC_50_ values obtained for compounds **2 e**–**i** revealed no direct correlation to the substituent's atomic radius: Interestingly, the activities of fluoro‐ and bromo‐substituted compounds were similar, while chloro‐substitution slightly improved the inhibition capacity, and iodo‐substitution reduced the affinity. These four halogenated derivatives all exert −I‐ and +M‐effects with different impacts depending on their electronegativity, thereby affecting their binding properties. Taking this into account, it seems that the unfavorable size of bromine is compensated by its weaker electron‐withdrawing properties. Vice versa, fluorine has favorable steric but unfavorable electronic properties. In this set of substituents, chlorine seems to have the best and iodine the worst combination of both. The relevance of the electron‐withdrawing effect is underlined when comparing the IC_50_ values of the bioisosteric substituents methyl (**2 a**) and trifluoromethyl (**2 d**), which are similar in size, but differ in their electronic properties. The moderate +I‐effect of the methyl substituent is contrasted by the impactful −I‐effect of the trifluoromethyl group resulting in a loss of binding affinity. Therefore, **2 d** is even less active than the unsubstituted **2 c**. In summary, in this tested set of compounds, the methyl group was the most beneficial substituent.

The exchange of the benzamide group displayed the relevance of this moiety for binding affinity. When phenyl (**2 c**) was substituted for aliphatic groups of similar size and/or lipophilicity (**2 j** and **2 k**), the inhibitors were inactive. The same holds true for sterically more demanding substituents: While benzyl (**2 l**) still showed some inhibition comparable to phenyl (**2 c**) in the screening, a branched diphenylmethane derivative (**2 m**) showed no inhibition, probably due to spatial limitations at the binding site. However, a bioisosteric exchange of benzene for thiophene (**2 n**) was possible with only a minor decrease in inhibition.

The effect of the amino group R^5^ at the benzamide moiety was evaluated by varying its position and substitution. Compared to **2 a**, the addition of this amino group increased the inhibitory capacity. Since **2 b** and **2 p** showed virtually identical inhibition, the impact of this modification seems to be independent of its position. Further derivatization of the amino group (regardless of the electronic influence) generally tended to negatively affect affinity, as illustrated by the dimethylamino‐ (**2 r**), sulfonamide‐ (**2 t**) and carbamate (**2 q**) derivatives, all of which showing reduced activity.

Compounds that showed activity as benzamides lost their activity when the amide groups were inverted to give the corresponding anilides (**11 a**–**d**), demonstrating the important role of the orientation of the linking amide between phenyl‐ and (naphthalen‐1‐yl)ethyl‐moieties. For synthetic reasons, only racemates of the anilides were assayed.

With the synthesis of isoindolines (Table 2) possessing a basic amine (calc. p*K*_a_ of the protonated species ca. 8.5), it was possible to design a new scaffold which, in contrast to the abovementioned inhibitors, has no amide group and is rigidified by cyclization. While isoindolinones and isoindolinediones were initially also considered as cyclic derivatives of the amide‐based inhibitors, those structures are lacking the H‐bond donor or charged functionality found in amides and isoindolines, respectively, to interact with Asp‐165. Therefore, isoindolinones and isoindolindiones were not followed up. Among the isoindolines, the unsubstituted **14 h** with an IC_50_ of 2.9±0.2 μM was the most promising compound. H‐bond donor substituents as ‐OH (**14 l)** and ‐NH_2_ (**14 k**) in the 5‐position caused only a slight reduction in affinity and showed similar inhibitory activities as **14 h**. Similar to the benzamide series above, inversion of the stereo center of the ethylnaphthylamine abolished activity (**14 i**). Interestingly, in contrast to the observations for benzamide‐type inhibitors, a 4‐methyl substitution at the aromatic moiety of the isoindoline ring system (**14 a**) was found to reduce inhibitory activity.

**Table 2:** Inhibition of SARS‐CoV PL^pro^ by isoindoline derivatives.

Compd	Structure	IC_50_ [μM]/%^[a]^	
		SARS‐CoV PL^pro^	SARS‐CoV‐2 PL^pro^
**20**	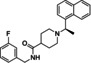	0.15±0.01^[21]^	
**14 a**	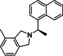	45 %^[c]^	n.a.^[b]^
**14 b**	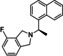	n.d. (insoluble)^[d]^	
**14 c^[e]^**	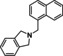	n.a.^[b]^	
**14 d**	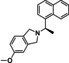	14±1	n.a.^[b]^
**14 e**	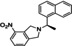	n.a.^[c]^	
**14 f**	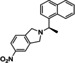	n.a.^[b]^	
**14 g**	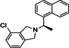	n.d. (insoluble)^[d]^	
**14 h^[e]^**	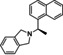	2.9±0.2	7.6±0.2
**14 i**	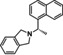	n.a.^[b]^	n.a.^[b]^
**14 j**	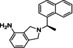	32±2	n.a.^[b]^
**14 k^[e]^**	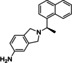	4.9±0.3	57±1
**14 l**	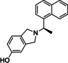	4.5±0.4	
**17**	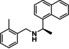	55 %

[a] The fluorometric assay was performed in duplicate. Data are reported as mean±standard deviation. Percentage inhibition was measured at 100 μM inhibitor concentration [b]. Compounds with inhibitory effects of less than 50 % at 100 μM compared to DMSO were labelled as not active (n.a.). [c] Percentage inhibition was measured at 10 μM inhibitor concentration with the addition of 0.005 % Brij 35. [d] IC_50_ values that could not be determined because of limited solubility at 100 μM under the conditions used in the assay are labelled as not determined (n.d.). [e] Compounds could only be obtained with a purity of 85–95 %. To complete the SAR study, the inhibitory effect was nevertheless tested.

The isoindolines have a positively charged amine function in close proximity to the (naphthalen‐1‐yl)ethyl moiety. This is also found in the previously described compound **20** ^[21]^ indicating similar binding modes which is supported by our docking studies (see below).

In contrast to **14 a**, **2 a** and **20** in which the degrees of freedom are limited by either the isoindoline, the amide or the piperidine structure, compound **17** constitutes a more flexible derivative. This modification, however, decreased the affinity, indicating the relevance of rigidity at this position.

### Antiviral activity against SARS‐CoV‐2

**Figure 1:**
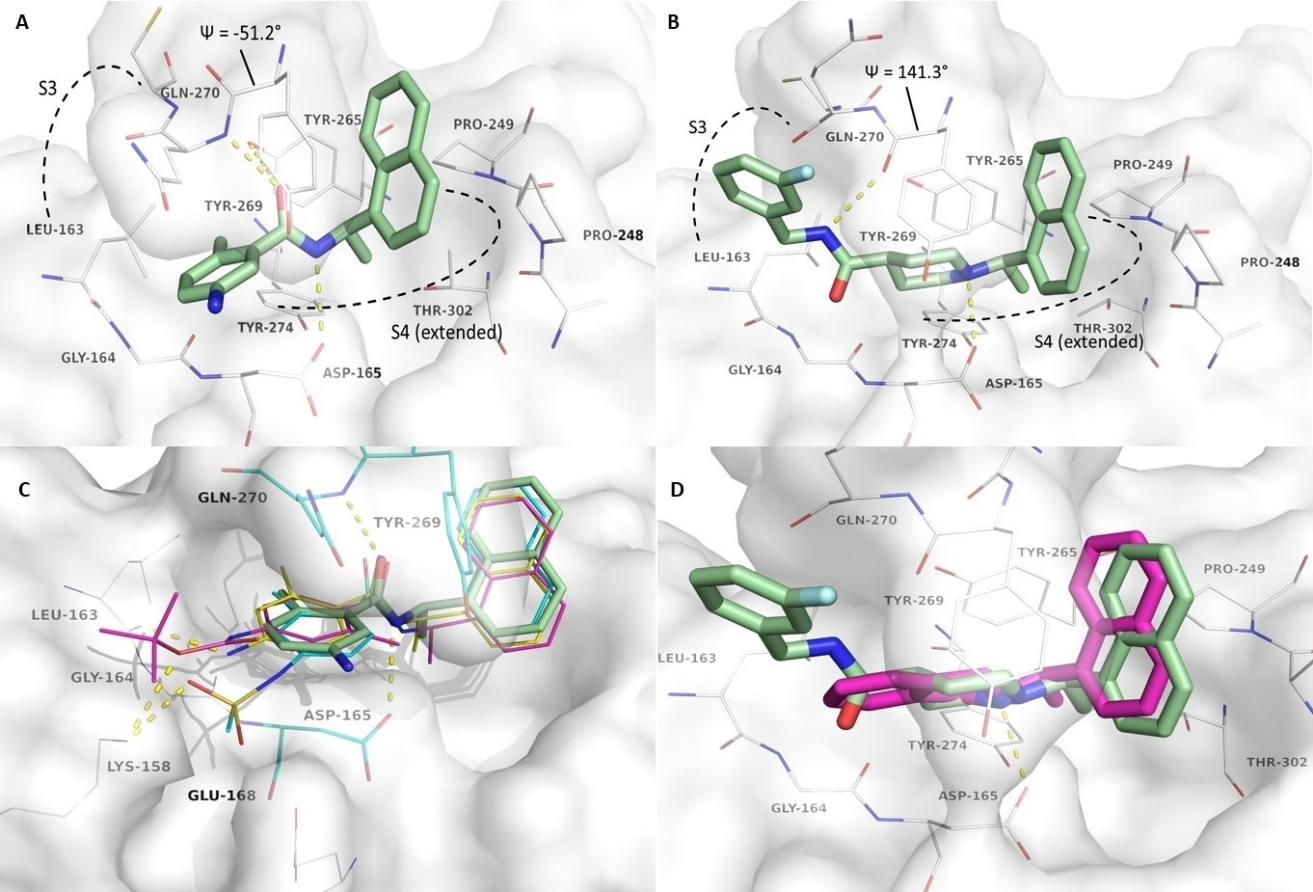
Crystallographic binding modes of **2 b** (**A**, PDB‐ID 3E9S) **13** and **20 (B**, PDB‐ID 4OW0)14 in complex with SARS‐CoV PL^pro^. Ligand atoms are depicted as sticks with green carbon atoms, protein with white carbon atoms and transparent surface. Polar interactions are indicated as yellow dashed lines. For a clear view, only residues with direct contacts to the ligands are labeled. (**C**) Predicted binding modes of **2 o** (magenta carbon atoms), **2 p** (yellow carbon atoms) and **2 s** (cyan carbon atoms) in complex with SARS‐CoV PL^pro^ (white carbon atoms, PDB‐ID 3E9S). Crystallographic reference ligand **2 b** is depicted as green sticks for orientation. (**D**) Predicted binding mode of **14 h** (magenta carbon atoms) in complex with SARS‐CoV PL^pro^ (white carbon atoms, PDB‐ID 4OW0). Crystallographic reference ligand **20** is depicted as green sticks for orientation.

A representative subset of both compound classes (benzamides and isoindolines) was tested for antiviral activity in cell culture using SARS‐CoV‐2‐infected (or mock‐infected) Vero E6 cells. Also, potential cytotoxicity was tested by a MTT assay. All compounds included in these experiments displayed low cytotoxicity (compare Table 3 and SI‐Figure 1), with CC_50_ values far above the respective EC_50_ values determined for these compounds.

**Table 3:** Inhibition of SARS‐CoV‐2 replication in Vero E6 cells and cytotoxicity in Vero E6 cells using a selection of benzamide and isoindoline inhibitors. Selectivity indices (SI) were calculated using the CC_50_ and EC_50_ values determined for the respective compounds.

Compd	Class	EC_50_ [μM]^[a]^	CC_50_ [μM]^[a]^	SI
**2 i**	Benzamide	1.77±1.60	376±1.36	212
**2 n**		4.74±1.35	>500	>105
**2 p**		15.75±1.59	>500	>31.7
**14 h**	Isoindoline	7.85±1.50	442±1.37	56.3
**14 k**		n.a.	>500	‐

[a] Experiments were performed in biological triplicates. Data are reported as mean±standard deviation.

The antiviral activities against SARS‐CoV‐2 of the benzamide type inhibitors in cell culture correlated quite well with their in vitro inhibition data determined for the SARS‐CoV PL^pro^. Similarly, consistent data were obtained for the isoindoline derivative **14 h**. In addition to the data presented in Table 3, other benzamide type inhibitors showed similar characteristics in preliminary SARS‐CoV‐2 inhibition experiments in cell culture (data not shown), demonstrating the major impact of reduced PL^pro^ activity on viral replication. With an EC_50_ value of 1.77 μM and a SI value of 212, the chloro‐substituted benzamide **2 i** was found to be one of the best PL^pro^ inhibitors of this series and we consider it the most promising compound for future studies.

Interestingly, **14 k**, which showed good activity in the fluorometric protease assay, was completely inactive in the viral replication assay. The reason for this behavior is not clear.

### Validation of SARS‐CoV SAR with SARS‐CoV‐2 data

In order to estimate the transferability of the SAR deduced from SARS‐CoV PL^pro^ to SARS‐CoV‐2 PL^pro^, a subset of compounds was tested in an *in vitro* protease assay against isolated SARS‐CoV‐2 PL^pro^.

It could be shown that compounds that are inactive against SARS‐CoV PL^pro^ did also not show inhibitory activity against SARS‐CoV‐2 PL^pro^. Examples for this are **4 d**, **11 a** and **14 i**. In general, the measured inhibitory effect against SARS‐CoV‐2 PL^pro^ was lower compared to SARS‐CoV PL^pro^. Some compounds that showed acceptable inhibition against SARS‐CoV PL^pro^ (i. e. **2 c**, **2 e**–**h**, **2 t**, **14 a**, **14 d**) were less active against SARS‐CoV‐2 PL^pro^. This is underlined by the determined IC_50_ values of **2 a**, **2 i**, **2 n**, **2 p**, **2 r**, and **2 s** against SARS‐CoV‐2 PL^pro^, that are higher than the respective values against SARS‐CoV PL^pro^ (Table 1, 2).

On the other hand, the correlation between the inhibition data obtained in the SARS‐CoV PL^pro^ AMC‐based enzyme‐activity assay and the SARS‐CoV‐2 antiviral assay is quite convincing. The reason for this discrepancy can be found in the differences in substrate affinities between the two homologous enzymes. The pentapeptidic AMC‐substrate that mimics the C‐terminus of ubiquitin (RLRGG) shows different K_M_‐values for both PL^pro^s.^[14]^ This results in differences in the competition between substrate and inhibitor, and as a result the AMC‐substrate is replaced much easier from SARS‐CoV PL^pro^ than from SARS‐CoV‐2 PL^pro^, yielding lower IC_50_ values for SARS‐CoV PL^pro^. Therefore, in the SARS‐CoV‐2 AMC‐based assay, higher inhibitor concentrations are necessary to achieve the same effects.

### Computational studies

Crystal structures of SARS‐CoV PL^pro^ in complex with naphthylethylamine‐substructure containing inhibitors were previously determined and are freely available in the protein data bank (PDB 3E9S, 4OW9).[^20^, ^21^, ^22^] Earlier reported and structurally closely related inhibitors address the S3 and (extended) S4 binding pocket adjacent to the active site and can be classified into basic piperidine‐based inhibitors (**20**)^[21]^ and neutral benzamide scaffolds (**2 b**).^[20]^ For both inhibitors, the β‐turn/loop (residues 267–272) is closed over the ligands by a proposed induced fit mechanism,^[23]^ by which the 1‐naphthyl‐moiety is packed in a hydrophobic pocket between Pro248, Pro249 and Tyr269 of the closed loop (Figure 1). The (*R*)‐methyl substituent is oriented toward a cavity forming hydrophobic interactions with Thr302, Tyr265 and Tyr274. Both ligands interact with Asp165 by either an H‐bond of the amide for **2 b** or an ionic interaction of the basic center in **20**. The oxygen atom of the amide in **2 b** further acts as an H‐bond acceptor for Tyr265 and the backbone of Gln270. However, this residue's donor is oriented away from the ligand in the PL^pro^‐**20** complex where Gln270 is flipped out of the pocket, which can be seen in the Ψ‐angle of Tyr269 (‐51° for the **2 b**‐complex and 141° in the **20**‐complex, respectively, compare Figure 1). This orientation allows an additional H‐bond to be formed by the ligand's amide donor toward the backbone oxygen of Tyr269 and offers space for an additional fluorophenyl‐substituent. For the small amide‐based inhibitor **2 b**, the Gln270 orientation allows close contacts of the phenyl ring with the surface areas of Gly164, Asp165 and Gln270. The methyl substituent is further oriented toward a small hydrophobic pocket shaped by Leu163, Tyr 265 and Tyr274.

For the interpretation of the observed SAR, a molecular docking protocol was established and validated by redocking and discrimination between binders and non‐binders, generating ROC curves with AUC values of 0.99 for benzamide‐type inhibitors in 3E9S and 0.89 for piperidine‐/isoindoline‐type inhibitors in 4OW0 (SI‐Figure 3). The naphthyl moiety seems to form the best shape complementarity within the hydrophobic pocket between Pro249, Pro248 and Tyr269 compared to smaller substituents like **4 a**. Further, the (*R*)‐configuration at the stereo‐center is preferred, most likely due to a steric clash with Tyr265 for the (*S)*‐isomer. While the methyl substituent at this stereo center is oriented toward a rather large sub‐pocket, larger hydrophobic substituents come along with a lower inhibitory potency ((**8**) and previously reported inhibitors^[21]^). This sub‐pocket is highly polar as it is formed by side chains of Asp165, Arg167, Tyr274, Thr302 and Asp303. Three water molecules (numbered 1035, 1040 and 1041 in PDB‐ID 4OW0) found in this pocket might be hard to be displaced due to their good coordination (SI‐Figure 4A).^[21]^ An inversion of the adjacent amide bond in compounds **11 a**–**d** is not tolerated due to the observed donor‐acceptor pattern with Asp165 and Gln270 (Figure 1A).

By derivatization of the 2‐substitution pattern of the phenyl ring, the size of the hydrophobic pocket at Leu163, Tyr265 and Tyr274 was explored. From crystal structures of SARS‐CoV PL^pro^ in complex with modified peptides (SI‐Figure 4B) it is known that Leu163 can be slightly displaced and offers space toward the active site.^[24]^ However, this might not to be expected in this series as halogen substituents with larger atomic volume revealed increased IC_50_‐values (**2 g**–**i**). This also accounts for trifluoromethyl‐ (**2 d**), nitrile‐ (**2 e**) and ethyl‐substituents (**19**).^[20]^ Chlorine or methyl derivatives with IC_50_‐values of 6.5 μM and 3.3 μM (**2 g** and **2 a**) can be assumed to be favorable in size, since an unsubstituted 2‐position with a gap in the hydrophobic interaction surface (**2 c**, IC_50_=62 μM) leads to lowered affinity. Modification of the phenyl moiety itself is not well tolerated (**2 j**–**m**), with only the bioisosteric replacement by thiophene (**2 n**) maintaining adequate potency. This trend was also observed in the docking studies, indicating the importance of a small aromatic system to form π‐π interactions with Tyr269 and hydrophobic interactions with CH_2_ of Asp165 and Gln270 (Figure 1A).

With compounds **2 o**–**t**, the influence of polar substituents was elucidated, revealing the most potent ligands. The polar substituents are oriented toward the solvent and additional polar interactions with the protease can be formed via H‐bonds with the Leu163 backbone oxygen (**2 o**–**q**) and the Lys158 side‐chain (**2 o**, **2 q**, **2 s**) (Figure 1C).

Finally, cyclic compounds **14 h**–**l** were designed to reduce the flexibility of the inhibitor. By this modification, the amide moiety is lost and a basic center is introduced which forms an ionic interaction with Asp165, comparable to piperidine‐containing inhibitors.^[21]^ While sterically and electrostatically all isoindoline‐inhibitors fit into the binding site (Figure 1D), the affinity of this ligand series highly relies on this ionic interaction and, thus, the basicity of the amine. Inactive compounds **14 e** and **14 f** with an electron‐withdrawing nitro group show lower calculated p*K*_a_‐values (Table 4). Accordingly, at physiological pH, the dominant protomer is the neutral form, which apparently does not bind to PL^pro^.

**Table 4:** Predicted basicity of isoindoline inhibitors and relative occurrence of positively charged protomer at pH=7.4 (%pp^7.4^), both calculated with MOE2019.01.

Compd	p*K*_a_	%pp^7.4^	Compound	p*K*_a_	%pp^7.4^
**14 a**	8.1	82 %	**14 h**	8.1	82 %
**14 b**	7.0	28 %	**14 i**	8.1	82 %
**14 c**	7.9	77 %	**14 j**	8.2	87 %
**14 d**	8.0	79 %	**14 k**	8.5	92 %
**14 e**	6.5	11 %	**14 l**	8.0	81 %
**14 f**	7.3	43 %	**17**	8.9	97 %
**14 g**	7.9	75 %

## Conclusion

In this work, a set of more than 40 inhibitors targeting the SARS‐CoV PL^pro^ was synthesized. Starting from the previously described inhibitor **2 a**, new compounds with antiproteolytic and antiviral effects in a single‐digit micromolar range were found. Additionally, the isoindoline moiety was identified as a promising new scaffold for PL^pro^ inhibitors.

Based on the synthesized derivatives, we determined SAR that can aid the future development of high‐affinity ligands. By varying the naphthylethylamine moiety, we could show its importance for inhibitory effects. The (*R*)‐configuration of naphthylethylamine is highly privileged which could be shown for both benzamides and isoindolines. Inversion of the amide bond, yielding anilide‐derivatives, resulted in loss of activity. Non‐aromatic residues as alternatives for the benzamide were not tolerated. By introducing different substituents in ortho position, we found the methyl‐substituted compound **2 a** to be most active followed by halogenated compounds, indicating a major role for CH/π‐interactions of the benzamide.

Variations of the amino‐substituent of compound **2 b** showed that the position of this substitution does not significantly impact the IC_50_ value.

With an IC_50_ value of 2.9±0.2 μM, the unsubstituted isoindoline inhibitor **14 h** is a promising starting point for further optimization, as it is likely to adopt a similar pose as **2 b** and gives space for further optimization.

Using molecular docking and scoring, a model was developed, providing a reliable tool to discriminate binders and non‐binders of PL^pro^. Moreover, binding modes of the inhibitors in the S3 and S4 pockets of the active site were predicted, elucidating important interactions.

Importantly, we were able to show the inhibitory capacity in cell culture of benzamide‐ and isoindoline‐type compounds against SARS‐CoV‐2, the causative agent of the current pandemic. The finding that **14 h** showed an EC_50_ value of 7.85 μM combined with its low cytotoxicity underlines its high potential for further development.

A representative subset of inhibitors showed inhibitory activity against isolated PL^pro^ from SARS‐CoV‐2, further validating the proposed mechanism of action against SARS‐CoV‐2. Together, these results lead us to the suggestion that these classes of inhibitors can be used in the development of broad‐spectrum PL^pro^‐inhibitors acting against this protease in related betacoronaviruses and, potentially, even other coronaviruses.

## Experimental Section

**Synthesis**. All commercially available reagents were obtained from Sigma Aldrich (Missouri, USA), Alfa AESAR (Massachusetts, USA), Fisher Scientific (New Hampshire, USA) and used without further purification except of 2,6‐dimethylaniline which was distilled under reduced pressure before storage under argon atmosphere. The commercially available solvents used for the synthesis were, if necessary, purified by distillation and desiccated by standard methods. Column chromatography was performed with silica gel (0.06–0.02 mm or 0.040–0.063 mm) obtained from Macherey‐Nagel (Düren, Germany). All reactions were monitored by thin‐layer chromatography using Macherey‐Nagel ALUGRAM Xtra SIL G/UV_254_ silica gel 60 plates for detection at 254 nm. For determination of melting points, a Stuart SMP10 (Cole‐Parmer, Stone, UK) or a Mettler FP51 (Mettler Toledo, Columbus, USA) was used. Measurements were performed in open capillaries; results were not corrected. All LC–MS measurements were performed on an Agilent 1100 system (Agilent Technologies, Santa Clara, USA) consisting of following components: degasser, bin pump, ALS, DAD, MSD Trap SL and a phenomenex Gemini 5 μ C18 110A (150×2.00 mm, 5 μM) column. Purity of compounds was determined as the ratio of compound's peak area to the sum of all peak areas in the UV spectra. The following methods were established to analyze the total amount of compounds (mobile phase A: 0.1 % formic acid in water, mobile phase B: 0.1 % formic acid in methanol): *method 5 % B* (flow rate: 0.3 mL/min, 0–10 min 5 % B, 10–13 min 100 % B), *method 5Bshort* (flow rate 0.4 mL/min, 0–10 min 5 % B, 10–13 min 100 % B), *method Gem3* (flow rate: 0.3 mL/min, 0–7 min 30 % B, 7–10 min 100 % B, *method Gem4* (flow rate: 0.4 mL/min, 0–5 min 60 % B, 5–8 min 100 % B), *method Gem5* (flow rate: 0.4 mL/min, 0–15 min 30 % B, 15–18 min 100 % B), *method Gem6* (flow rate: 0.3 mL/min, 0–15 min 50 % B, 15–18 min 100 % B), *method Gem7* (flow rate: 0.3 mL/min, 0–12 min 15 % B, 12–13 min 70 % B, 13–16 min 100 % B), *method Gem8* (flow rate: 0.3 mL/min, 0–10 min 65 % B, 10–13 min 100 % B). ^1^H, ^13^C, spectra were measured on a Bruker Avance 400 (400 MHz) spectrometer (Bruker, Billerica, USA) and a Varian Mercury 400 (400 MHz) spectrometer (Agilent Technologies, Santa Clara, USA). For assignments DEPT, HHCOSY, HMQC and HMBC experiments were performed. Chemical shifts are reported in parts per million (ppm). Signals of solvents were set as lock substance and reference for chemical shifts, multiplicity is indicated by the following abbreviations (br=broad, s=singlet, d=doublet, t=triplet, q=quartet, dd=doublet of doublet, ddd=doublet of doublet of doublet, dddd=doublet of doublet of doublet of doublet). Signal assignments of protons and carbon cores were conducted by 2D correlation spectra.

**Aa/Ab General procedure for the synthesis of amides from carboxylic acid chlorides in a two‐phase system**. The amine (1.0 eq.) was dissolved in Et_2_O (50 mL) and 10 mL 10 % sodium carbonate solution were added. The acid chloride (1.1–1.3 eq.) was added under vigorous stirring.

Amides which were poorly soluble in Et_2_O were filtered and dried. Soluble amides were extracted with ethyl acetate (10 mL). The organic phase was washed with semi‐saturated sodium bicarbonate solution, dried over sodium sulfate and the solvent was removed.

**Ac General procedure for the synthesis of amides from carboxylic acid chlorides in a single phase system**. The amine (≥2.0 eq.) was dissolved in THF (5 mL) and the carboxylic acid chloride (1.0 eq.) was added dropwise under vigorous stirring. After 2 d, 0.05 M hydrochloric acid (30 mL) was added and the mixture was extracted by ethyl acetate (30 mL). The organic phase was washed with sodium hydrogen carbonate solution followed by saturated sodium chloride solution and dried over sodium sulfate. The solvent was evaporated, and the crude product recrystallized from an appropriate solvent.

**B General procedure for the synthesis of amides catalyzed by DCC**. The carboxylic acid (1.0 eq.) and HOBt‐hydrate (1.1 eq.) were dissolved in an appropriate solvent. The reaction mixture was cooled to 0 °C and DCC (1.1 eq.) was added. The reaction was warmed to r.t. and the amine (1.0 eq.) was added. The reaction mixture was stirred at r.t. for 1 d and precipitated dicyclohexylurea was filtered off. The filtrate was reduced *in vacuo* and the crude product was purified by silica gel column chromatography.

**C General procedure for the synthesis of amides catalyzed by DIC**. The carboxylic acid (1.2 eq) and HOBt‐hydrate (1.7 eq.) were dissolved in THF. The mixture was cooled to 0 °C and DIC (1.2 eq.) was added dropwise. After 30 min, the amine (1.0 eq.) was added and the reaction mixture was warmed to r.t.. After stirring overnight, the solvent was evaporated. The residue was taken up in ethyl acetate (5 mL) and washed three times with 10 % sodium carbonate solution. The organic phase was dried over sodium sulfate and the solvent was evaporated.

**D General procedure for the synthesis of isoindolines by dialkylation of primary amines**. The primary amine (1.0 eq.), TEA (≥2.0 eq.) and the *bis*(bromomethyl)benzene derivative (1.0–1.2 eq.) were dissolved in toluene (30 mL) and heated one hour under reflux. The solvent was evaporated, and the residue was taken up in ethyl acetate (30 mL). The solution was washed twice with 10 % sodium carbonate solution (30 mL) followed by saturated sodium chloride solution (30 mL). The organic phase was dried over sodium sulfate and the solvent was evaporated. The crude product was purified by silica gel column chromatography (petroleum ether/ethyl acetate 4 : 1). The resulting isoindoline was taken up in HCl in methanol (4 M, 1 mL) and the solvent was evaporated under reduced pressure.

**E General procedure for the reduction of** ***N*** **‐alkylated phthalimide derivatives**. The phthalimide derivative (1.0 eq.) was dissolved in THF abs. (20 mL) under argon atmosphere. LAH (3–5 eq.) was added to the solution. The mixture was heated under reflux for 2 h and then cooled to r.t.. Excess LAH was quenched by a few drops of ethanol and water and the mixture was mixed with potassium carbonate solution (2 mL). The formed precipitate was separated and washed with THF. The filtrate was reduced *in vacuo* and the residue was dissolved in ethyl acetate. The solution was washed with 0.1 M sodium hydroxide solution (20 mL) followed by saturated sodium chloride solution (10 mL). The organic phase was dried over sodium sulfate and the solvent was evaporated. The crude product was purified by silica gel column chromatography (petroleum ether/ethyl acetate 4 : 1).

**F General procedure for the reduction of aromatic nitro groups**. The aromatic nitro derivative (1.0 eq.) and stannous(II) chloride (3.0 eq.) were refluxed in semi‐concentrated hydrochloric acid (20 mL) for 2 h. After cooling to r.t., the mixture was extracted with diethyl ether and the organic phase was discarded. The aqueous phase was adjusted to pH>10 by 20 % sodium hydroxide solution. The solution was extracted three times with ethyl acetate and the combined organic phases were dried over sodium sulfate. The solvent was evaporated, and the product kept under argon atmosphere.

**G General procedure for the removal of Boc‐protecting groups**. This compound was synthesized according to the general procedure E. The Boc‐protected compound (1 eq.) was dissolved in methanol and 4 m methanolic hydrochloric acid (1 mL, 11.5 eq.) was added. After 2 h, the solvent was evaporated under reduced pressure to yield the product which did not need further purification.

**2‐Methyl‐*N*‐[(1*R*)‐naphth‐1‐ylethyl]benzamide (2 a):^[20]^** This compound was synthesized according to the general procedure Ac. 394 mg, 1.36 mmol, yield: 43 %. mp: 160 °C. R_f_=0.48 (CH/EtOAc 3 : 1). ^1^H NMR (400 MHz, CDCl_3_) δ=1.81 (d, *J*=6.82 Hz, 3H), 2.45 (s, 3H), 5.97 (d, *J*=7.33 Hz, 1H), 6.06–6.23 (m, 1H), 7.09–7.22 (m, 2H), 7.23–7.32 (m, 2H), 7.41–7.63 (m, 4H), 7.83 (d, *J*=8.08 Hz, 1H), 7.87–7.92 (m, 1H), 8.26 (d, *J*=8.34 Hz, 1H) ppm. ^13^C NMR (100 MHz, CDCl_3_) δ=19.7, 20.6, 44.9, 122.6, 123.6, 125.2, 125.7, 126.0, 126.5, 126.6, 128.5, 128.8, 129.8, 130.9, 131.2, 134.0, 136.1, 136.4, 138.0, 169.0 ppm. MS (ESI): *m/z* calcd for C_20_H_19_NO [M+H]^+^290.15, found 290.3. Purity (LC, 300 nm):>99 %.

(***R*** **)‐5‐Amino‐2‐methyl‐*N*‐[1‐(naphth‐1‐yl)ethyl]benzamide hydrochloride (2 b):^[20]^** This compound was synthesized according to the general procedure G. 92 mg, 0.27 mmol, yield: 99 %. R_f_=0.18 (CH/EtOAc 1 : 1), ^1^H NMR (400 MHz, MeOD) δ=1.73 (d, *J*=6.88 Hz, 3H), 2.37 (s, 3H), 6.07 (q, *J*=6.88 Hz, 1H), 7.28–7.44 (m, 3H), 7.45–7.62 (m, 3H), 7.65 (d, *J*=7.15 Hz, 1H), 7.82 (d, *J*=8.28 Hz, 1H), 7.91 (d, *J*=7.65 Hz, 1H), 8.25 (d, *J*=8.41 Hz, 1H) ppm. ^13^C NMR (100 MHz, MeOD) δ=19.3, 21.4, 46.6, 122.8, 124.0, 124.3, 125.3, 126.6, 126.9, 127.5, 129.3, 129.6, 130.1, 132.5, 133.6, 135.6, 138.2, 140.0, 140.1, 170.2 ppm. MS (ESI): m/z calcd for C_20_H_21_ClN_2_O [M+H]^+^305.17, found 305.0. Purity (LC, 300 nm): 95.5 %.

***N*** **‐[(1*R*)‐1‐Naphth‐1‐ylethyl]benzamide (2 c)**: This compound was synthesized according to the general procedure B. 34 mg, 0.12 mmol, yield: 38 %. mp: 195 °C. R_f_=0.4 (CH/EtOAc 3 : 1). ^1^H NMR (400 MHz, CDCl_3_) δ=1.80 (d, *J*=6.78 Hz, 3H), 6.06–6.21 (m, 1H), 6.40 (d, *J*=7.53 Hz, 1H), 7.35–7.43 (m, 2H), 7.43–7.58 (m, 4H), 7.61 (d, *J*=7.15 Hz, 1H), 7.70–7.78 (m, 2H), 7.83 (d, *J*=8.16 Hz, 1H), 7.86–7.92 (m, 1H), 8.19 (d, *J*=8.28 Hz, 1H) ppm. ^13^C NMR (100 MHz, CDCl_3_) δ=20.6, 45.2, 122.7, 123.4, 125.2, 125.9, 126.7, 126.9, 128.5, 128.8, 131.2, 131.4, 134.0, 134.5, 138.1, 166.4 ppm. MS (ESI): m/z calcd for C_19_H_17_NO [M+H]^+^276.14, found 276.2. Purity (LC, 254 nm): 98.6 %.

***N*** **‐[(1*R*)‐1‐Naphth‐1‐ylethyl]‐2‐(trifluormethyl)benzamide (2 d)**: Trifluoromethylbenzoic acid (237 mg, 1.25 mmol, 1 eq.), PPA in ethyl acetate (792 mg, 1.25 mmol, 1 eq.), (1*R*)‐1‐naphth‐1‐ylethylamine (200 μL, 1.25 mmol, 1 eq.) and NMM (176 mg, 2.72 mmol, 2.2 eq.) were successively added to dichloromethane and stirred overnight at r.t.. The reaction mixture was diluted with dichloromethane (30 mL) and washed with 10 % sodium carbonate solution (3x 20 mL) followed by saturated sodium chloride. The organic phase was dried over sodium sulfate and the solvent was evaporated under reduced pressure. The crude product was recrystallized from dichloromethane/petroleum ether 1 : 2 to yield **2 d** (168 mg, 0.489 mmol, yield: 39 %). mp: 161 °C. ^1^H NMR (400 MHz, CDCl_3_) δ=1.81 (d, *J*=6.57 Hz, 3H), 5.99 (d, *J*=7.58 Hz, 1H), 6.07–6.23 (m, 1H), 7.40–7.65 (m, 7H), 7.65–7.74 (m, 1H), 7.83 (d, *J*=8.34 Hz, 1H), 7.86–7.94 (m, 1H), 8.24 (d, *J*=8.59 Hz, 1H) ppm. ^13^C NMR (100 MHz, CDCl_3_) δ=20.0, 45.3, 122.8, 123.4, 125.0, 125.2, 126.0, 126.3, 126.7, 127.1, 128.5, 128.7, 128.8, 129.8, 132.4, 131.9, 135.8, 135.8, 137.5, 166.6 ppm. MS (ESI): m/z calcd for C_20_H_16_F_3_NO [M+H]^+^344.13, found 344.2. Purity (LC, 254 nm): 98.7 %.

**(*R*)‐2‐Cyano‐*N*‐(1‐(naphth‐1‐yl)ethyl)benzamide (2 e)**: This compound was synthesized according to the general procedure C. 90 mg, 0.30 mmol, yield: 48 %. mp: 181 °C. ^1^H NMR (400 MHz, CDCl_3_) δ=1.83 (d, *J*=6.82 Hz, 3H), 6.15 (qd, *J*=7.07, 6.82 Hz, 1H), 6.56 (d, *J*=7.07 Hz, 1H), 7.41–7.66 (m, 6H), 7.66–7.77 (m, 2H), 7.82 (d, *J*=8.34 Hz, 1H), 7.88 (d, *J*=8.08 Hz, 1H), 8.20 (d, *J*=8.34 Hz, 1H) ppm. ^13^C NMR (100 MHz, CDCl_3_) δ=20.6 (*C*H3), 45.9 (*C*H), 110.4, 117.5, 122.9, 123.3, 125.3, 126.0, 126.8, 128.6, 128.7, 128.8, 130.9, 131.0, 132.8, 133.9, 133.9, 137.6, 138.5, 164.2 ppm. MS (ESI): m/z calcd for C_20_H_16_N_2_O [M+H]^+^301.14, found 301.1. Purity (LC, 254 nm): 98.7 %.

**2‐Fluor‐*N*‐[(1*R*)‐1‐naphth‐1‐ylethyl]benzamide (2 f)**: This compound was synthesized according to the general procedure C. 115 mg, 0.39 mmol, yield: 63 %. mp: 135 °C. R_f_=0.45 (CH/EtOAc 3 : 1). ^1^H NMR (400 MHz, CDCl_3_) δ=1.80 (d, *J*=6.82 Hz, 3H), 6.16–6.25 (m, 1H), 7.00–7.09 (m, 1H), 7.09–7.18 (m, 1H), 7.24 (td, *J*=7.58, 1.01 Hz, 1H), 7.42 (dddd, *J*=8.27, 7.33, 5.24, 1.89 Hz, 1H), 7.46–7.65 (m, 4H), 7.82 (d, *J*=8.08 Hz, 1H), 7.86–7.93 (m, 1H), 8.12 (td, *J*=7.83, 1.77 Hz, 1H), 8.23 (d, *J*=8.34 Hz, 1H) ppm. ^13^C NMR (100 MHz, CDCl_3_) δ=21.1, 45.4, 115.8, 121.1, 122.5, 123.2, 124.6, 125.2, 125.7, 126.4, 128.2, 128.7, 130.9, 131.9, 133.1, 133.9, 138.3, 160.5, 162.2 ppm MS (ESI): m/z calcd for C_19_H_16_FNO [M+H]^+^294.13, found 294.1. Purity (LC, 254 nm): 97.5 %.

**2‐Bromo‐*N*‐[(1*R*)‐1‐naphth‐1‐ylethyl]benzamide (2 g)**: This compound was synthesized according to the general procedure C. 120 mg, 0.34 mmol, yield: 55 %. mp: 153 °C. ^1^H NMR (400 MHz, CDCl_3_) δ=1.83 (d, *J*=6.53 Hz, 3H), 6.10–6.19 (m, 1H), 6.19–6.26 (m, 1H), 7.20–7.26 (m, 1H), 7.30 (td, *J*=7.47, 1.25 Hz, 1H), 7.45–7.63 (m, 6H), 7.83 (d, *J*=8.16 Hz, 1H), 7.86–7.92 (m, 1H), 8.26 (d, *J*=8.66 Hz, 1H) ppm. ^13^C NMR (100 MHz, CDCl_3_) δ=20.5, 45.5, 119.3, 122.8, 123.6, 125.2, 125.9, 126.6, 127.5, 128.6, 128.8, 129.4, 131.1, 131.2, 133.3, 133.9, 137.6, 137.8, 166.5 ppm. MS (ESI): m/z calcd for C_19_H_16_BrNO [M+H]^+^354.05 (for ^79^Br), found 354.1 and 356.0. Purity (LC, 254 nm):>99 %.

**2‐Iodo‐*N*‐[(1*R*)‐1‐naphth‐1‐ylethyl]benzamide (2 h)**: This compound was synthesized according to the general procedure C. 76 mg, 0.19 mmol, yield: 31 %. mp: 161 °C. ^1^H NMR (400 MHz, CDCl_3_) δ=1.85 (d, *J*=6.78 Hz, 3H), 6.01 (d, *J*=7.65 Hz, 1H), 6.08–6.20 (m, 1H), 7.05 (ddd, *J*=8.03, 6.34, 2.82 Hz, 1H), 7.28–7.35 (m, 2H), 7.48 (dd, *J*=8.03, 7.40 Hz, 1H), 7.50–7.57 (m, 1H), 7.58–7.63 (m, 2H), 7.80–7.91 (m, 3H), 8.28 (d, *J*=8.41 Hz, 1H) ppm. ^13^C NMR (100 MHz, CDCl_3_) δ=20.3, 45.4, 92.4, 122.8, 123.7, 125.1, 126.0, 126.7, 128.1, 128.2, 128.6, 128.8, 131.1, 131.2, 133.9, 137.5, 139.9, 142.0, 168.2 ppm. MS (ESI): m/z calcd for C_19_H_16_INO [M+H]^+^402.04, found 402.0. Purity (LC, 254 nm): 93.8 %.

**2‐Chloro‐*N*‐[(1*R*)‐1‐naphth‐1‐ylethyl]benzamide (2 i)**: This compound was synthesized according to the general procedure Aa/Ab. 138 mg, 0.45 mmol, yield: 64 %. mp: 133 °C. R_f_=0.33 (CH/EtOAc 3 : 1). ^1^H NMR (400 MHz, CDCl_3_) δ=1.82 (d, *J*=6.78 Hz, 3H), 6.09–6.23 (m, 1H), 6.42 (d, *J*=7.28 Hz, 1H), 7.22–7.40 (m, 3H), 7.44–7.64 (m, 5H), 7.82 (d, *J*=8.28 Hz, 1H), 7.86–7.94 (m, 1H), 8.24 (d, *J*=8.53 Hz, 1H) ppm. ^13^C NMR (100 MHz, CDCl_3_) δ=20.6, 45.6, 122.7, 123.6, 125.2, 125.9, 126.6, 127.0, 128.5, 128.8, 130.0, 130.2, 130.7, 131.1, 131.2, 134.0, 135.2, 137.8, 165.5 ppm. MS (ESI): m/z calcd for C_19_H_16_ClNO [M+H]^+^310.1, found 310.2. Purity (LC, 254 nm): 98 %.

***N*** **‐[(1*R*)‐1‐Naphth‐1‐ylethyl]cyclohexanecarboxamide (2 j)**: This compound was synthesized according to the general procedure C. 93 mg, 0.33 mmol, yield: 53 %. mp: 170 °C. R_f_=0.51 (CH/EtOAc 3 : 1). ^1^H NMR (400 MHz, CDCl_3_) δ=1.16–1.65 (m, 6H), 1.67 (d, *J*=6.57 Hz, 3H), 1.70–1.91 (m, 4H), 2.04 (tt, *J*=11.75, 3.41 Hz, 1H), 5.64 (d, *J*=7.58 Hz, 1H), 5.79–6.04 (m, 1H), 7.43–7.58 (m, 4H), 7.81 (d, *J*=8.08 Hz, 1H), 7.84–7.91 (m, 1H), 8.08 (d, *J*=8.34 Hz, 1H) ppm. ^13^C NMR (100 MHz, CDCl_3_) δ=20.6, 25.7, 25.7, 25.7, 29.6, 29.7, 44.2, 45.6, 122.5, 123.6, 125.1, 125.9, 126.5, 128.3, 128.7, 131.2, 133.9, 138.4, 174.8 ppm. MS (ESI): m/z calcd for C_19_H_23_NO [M+H]^+^282.19, found 282.2. Purity (LC, 254 nm): 98.3 %.

**(*R*)‐*N*‐(1‐Naphth‐1‐yl)ethyl)pivalamide (2 k)**: This compound was synthesized according to the general procedure Aa/Ab. 74 mg, 0.29 mmol, yield: 47 %. mp: 131 °C. ^1^H NMR (400 MHz, CDCl_3_) δ=1.18 (s, 9 H), 1.67 (d, *J*=6.53 Hz, 3H), 5.83 (d, *J*=6.65 Hz, 1H), 5.87–5.95 (m, 1H), 7.43–7.57 (m, 4H), 7.81 (d, *J*=7.91 Hz, 1H), 7.84–7.91 (m, 1H), 8.04–8.09 (m, 1H) ppm. ^13^C NMR (100 MHz, CDCl_3_) δ=20.5, 27.5, 38.6, 44.5, 122.4, 123.5, 125.1, 125.8, 126.4, 128.3, 128.7, 131.2, 133.9, 138.5, 177.2 ppm. MS (ESI): m/z calcd for C_17_H_21_NO [M+H]^+^256.17, found 256.2. Purity (LC, 254 nm):>99 %.

***N*** **‐[(1*R*)‐1‐Naphth‐1‐ylethyl]‐2‐phenylacetamid (2 l)**: This compound was synthesized according to the general procedure Aa/Ab. 150 mg, 0.52 mmol, yield: 84 %. mp: 130 °C. ^1^H NMR (400 MHz, CDCl_3_) δ=1.50 (d, *J*=6.78 Hz, 3H), 3.47 (d, *J*=1.76 Hz, 2H), 5.63 (d, *J*=7.15 Hz, 1H), 5.78–5.87 (m, 1H), 7.10–7.33 (m, 7H), 7.37–7.45 (m, 2H), 7.68 (d, *J*=8.03 Hz, 1H), 7.74–7.78 (m, 1H), 7.93–7.98 (m, 1H) ppm. ^13^C NMR (100 MHz, CDCl_3_) δ=20.8, 43.8, 44.9, 122.3, 123.4, 125.1, 125.8, 126.4, 127.2, 128.3, 128.7, 128.9, 129.3, 131.0, 133.9, 134.8, 138.2, 169.8 ppm. MS (ESI): m/z calcd for C_20_H_19_NO [M+H]^+^290.16, found 290.2. Purity (LC, 254 nm): 98.3 %.

**(*R*)‐*N*‐(1‐(naphthalen‐1‐yl)ethyl)‐2,2‐diphenylacetamide (2 m)**: This compound was synthesized according to the general procedure Aa/Ab. 168 mg, 0.46 mmol, yield: 73 %. mp: 152 °C. ^1^H NMR (400 MHz, CDCl_3_) δ=1.59 (d, *J*=6.78 Hz, 3H), 4.90 (s, 1H), 5.97 (qd, *J*=7.17, 6.96 Hz, 1H), 6.19 (d, *J*=5.65 Hz, 1H), 7.18–7.36 (m, 11H), 7.40 (t, *J*=7.59 Hz, 1H), 7.46–7.57 (m, 2H), 7.79 (d, *J*=8.03 Hz, 1H), 7.83–7.91 (m, 1H), 8.01–8.14 (m, 1H) ppm. ^13^C NMR (100 MHz, CDCl_3_) δ=20.5, 44.9, 58.6, 122.3, 123.3, 124.9, 125.6, 126.2, 126.9, 127.0, 128.0, 128.4, 128.4, 128.5, 128.7, 128.7, 130.8, 133.7, 138.1, 139.2, 170.6. MS (ESI): m/z calcd for C_19_H_16_ClNO [M+H]^+^365.18, found 366.3. Purity (LC, 254 nm): 96.4 %.

**3‐Methyl‐*N*‐[(1*R*)‐1‐naphth‐1‐ylethyl]thiophene‐2‐carboxamide (2 n)**: To a solution of 3‐methylthiophene‐2‐carboxylic acid (88.8 mg, 0.625 mmol, 1 eq.) and HBTU (237 mg, 0.625 mmol, 1 eq.) in DMF (2 mL), TEA (63.5 μL, 0.615 mmol, 1 eq.) was added dropwise. (1*R*)‐1‐Naphth‐1‐ylethanamine (100 μL, 0.624 mmol, 1 eq.) was added after 5 min. After stirring overnight at r.t., the reaction mixture was diluted with water (8 mL) and stirred again overnight. The resulted precipitate was filtered off and washed with 5 % ammonia solution (3x 5 mL) and dried to yield 152n (117 mg, 0.40 mmol, yield: 63 %). mp: 120 °C. R_f_=0.82 (CH/EtOAc 1 : 1). ^1^H NMR (400 MHz, CDCl_3_) δ=1.78 (d, *J*=6.65 Hz, 3H), 2.48 (s, 3H), 5.97–6.15 (m, 2H), 6.87 (d, *J*=4.89 Hz, 1H), 7.24 (d, *J*=5.02 Hz, 1H), 7.45–7.61 (m, 4H), 7.83 (d, *J*=8.16 Hz, 1H), 7.87–7.91 (m, 1H), 8.19 (d, *J*=8.53 Hz, 1H) ppm. ^13^C NMR (100 MHz, CDCl_3_) δ=15.7, 21.0, 45.3, 122.6, 123.4, 125.2, 125.9, 126.3, 126.6, 128.5, 128.8, 130.9, 131.1, 132.0, 134.0, 138.2, 141.2, 162.1 ppm. (ESI): *m/z* calcd for C_18_H_17_NOS [M+H]^+^296.11, found 296.1 Purity (LC, 254 nm):>99 %.

**(*R*)‐*tert*‐Butyl(3‐Methyl‐4‐((1‐(naphth‐1‐yl)ethyl)carbamoyl)phenyl)carbamate (2 o)**: This compound was synthesized according to the general procedure B. 200 mg, 0.49 mmol, yield: 62 %. R_f_=0.65 (PE/EtOAc 1 : 1). ^1^H NMR (400 MHz, CDCl_3_) δ=1.51 (s, 9H), 1.79 (d, *J*=6.82 Hz, 3H), 2.43 (s, 3H), 5.97 (d, *J*=8.34 Hz, 1H), 6.06–6.17 (m, 1H), 6.51 (s., 1H), 7.11 (dd, *J*=8.34, 2.02 Hz, 1H), 7.18–7.25 (m, 2H), 7.42–7.62 (m, 4H), 7.82 (d, *J*=8.08 Hz, 1H), 7.85–7.92 (m, 1H), 8.23 (d, *J*=8.59 Hz, 1H) ppm. MS (ESI): m/z calcd for C_25_H_28_N_2_O_3_ [M+H]^+^405.22, found 405.3. Purity (LC, 254 nm): 99.1 %.

**(*R*)‐4‐Amino‐2‐methyl‐*N*‐[1‐(naphth‐1‐yl)ethyl]benzamide hydrochloride (2 p)**: This compound was synthesized according to the general procedure G. 116 mg, 0.34 mmol, yield: 97 %. mp: 231 °C. R_f_=0.21 (CH/EtOAc 1 : 1). ^1^H NMR (400 MHz, MeOD) δ=1.72 (d, *J*=6.97 Hz, 3H), 2.39 (s, 3H), 6.06 (q, *J*=6.97 Hz, 1H), 7.21–7.29 (m, 2H), 7.43–7.66 (m, 5H), 7.82 (d, *J*=8.16 Hz, 1H), 7.91 (dd, *J*=8.41, 1.00 Hz, 1H), 8.25 (d, *J*=8.53 Hz, 1H) ppm. ^13^C NMR (100 MHz, MeOD) δ=19.7, 21.4, 46.6, 121.5, 123.9, 124.4, 126.1, 126.6, 126.9, 127.5, 129.3, 130.1, 130.1, 132.5, 133.1, 135.7, 139.2, 139.8, 140.2, 170.6 ppm. MS (ESI): m/z calcd for C_20_H_21_ClN_2_O [M+H]^+^305.17, found 305.2. Purity (LC, 300 nm): 99.0 %.

**(*R*)‐*tert*‐Butyl (4‐Methyl‐3‐((1‐(naphth‐1‐yl)ethyl)carbamoyl)phenyl) carbamate (2 q)**: This compound was synthesized according to the general procedure B. 114 mg, 0.28 mmol, yield: 70 %. R_f_=0.6 (PE/EtOAc 2 : 1). ^1^H NMR (400 MHz, CDCl_3_) δ=1.48 (s, 9H), 1.80 (d, *J*=6.40 Hz, 3H), 2.36 (s, 3H), 6.02–6.19 (m, 2H), 7.10 (d, *J*=8.28 Hz, 1H), 7.19–7.34 (m, 2H), 7.43–7.65 (m, 4H), 7.82 (d, *J*=8.16 Hz, 1H), 7.85–7.93 (m, 1H), 8.02 (s, 1H), 8.23 (d, *J*=8.53 Hz, 1H) ppm. MS (ESI): m/z calcd for C_25_H_28_N_2_O_3_ [M+H]^+^405.22, found 405.3. Purity (LC, 254 nm): 99.0 %.

**(*R*)‐5‐(Dimethylamino)‐2‐methyl‐*N*‐[1‐(naphth‐1‐yl)ethyl]benzamide (2 r)**: (*R*)‐5‐Amino‐methyl‐*N*‐[1‐(naphth‐1‐yl)ethyl]benzamide hydrochloride **12** (66 mg, 0.19 mmol, 1 eq.), methyl iodide (48 μL, 110 mg, 0.77 mmol, 4 eq.) and potassium carbonate (1.0 g, 7.2 mmol, 38 eq.) were dissolved in ethyl acetate and the solution was heated under reflux for 2 d. The reaction mixture was then washed with 10 % sodium carbonate (3x 10 mL). The organic phase was dried over sodium sulfate and the solvent was evaporated under reduced pressure. The crude product was purified by silica gel column chromatography (PE/EtOAc 3 : 1) to yield **2 r** (10 mg, 0.03 mmol, yield: 16 %). mp: 130 °C. R_f_=0.21 (CH/EtOAc 3 : 1). ^1^H NMR (400 MHz, CDCl_3_) δ=1.80 (d, *J*=6.78 Hz, 3H), 2.22–2.38 (m, 3H), 2.86 (s, 6H), 5.99 (d, *J*=7.91 Hz, 1H), 6.07–6.20 (m, 1H), 6.64–6.75 (m, 2H), 7.03 (d, *J*=8.16 Hz, 1H), 7.43–7.62 (m, 4H), 7.82 (d, *J*=8.03 Hz, 1H), 7.86–7.91 (m, 1H), 8.26 (d, *J*=8.53 Hz, 1H) ppm. ^13^C NMR (100 MHz, CDCl_3_) δ=18.5, 20.7, 40.8, 45.0, 111.3, 114.5, 122.6, 123.2, 123.6, 125.1, 125.9, 126.5, 128.4, 128.8, 131.2, 131.5, 134.0, 137.0, 138.2, 148.7, 169.6 ppm. MS (ESI): m/z calcd for C_22_H_24_N_2_O [M+H]^+^3.20, found 333.2. Purity (LC, 300 nm): 93.0 %.

**2‐Methyl‐4‐[(methylsulfonyl)amino]‐*N*‐[(1*R*)‐1‐naphth‐1‐ylethyl]benzamide (2 s)**: This compound was synthesized according to a modified general procedure Ab with (8 eq.) of methane sulfonyl chloride. 14 mg (0.04, yield: 11 %. mp: 166 °C. R_f_=0.22 (PE/EtOAc/FA 1 : 1:0.05). ^1^H NMR (400 MHz, CDCl_3_) δ=1.79 (d, *J*=6.65 Hz, 3H), 2.37 (s, 3H), 2.93 (s, 3H), 6.05–6.14 (m, 1H), 6.18 (d, *J*=8.28 Hz, 1H), 6.89–7.01 (m, 2H), 7.20 (d, *J*=8.16 Hz, 1H), 7.26 (s., 1H), 7.41–7.63 (m, 4H), 7.80 (d, *J*=8.28 Hz, 1H), 7.84–7.91 (m, 1H), 8.19 (d, *J*=8.28 Hz, 1H) ppm. ^13^C NMR (100 MHz, CDCl_3_) δ=19.9, 20.6, 39.4, 45.2, 116.8, 121.9, 122.6, 123.3, 125.2, 126.0, 126.6, 128.2, 128.6, 128.85, 131.1, 132.7,133.9, 137.8, 138.2, 138.3, 168.6 ppm. MS (ESI): m/z calcd for C_21_H_22_N_2_O_3_S [M+H]^+^383.15, found 383.6. Purity (LC, 254 nm):>99 %.

**2‐Methyl‐5‐(methylsulfamoyl)‐*N*‐[(1*R*)‐1‐naphth‐1‐ylethyl]benzamide (2 t)**: This compound was synthesized according to the general procedure B. 28 mg, 0.073 mmol, yield: 28 %. mp: 184 °C. ^1^H NMR (400 MHz, MeOD) δ=1.72 (d, *J*=6.90 Hz, 3H), 2.40 (s, 3H), 2.49 (s, 3H), 6.07 (q, *J*=6.90 Hz, 1H), 7.39–7.66 (m, 5H), 7.71–7.78 (m, 2H), 7.82 (d, *J*=8.16 Hz, 1H), 7.91 (d, *J*=7.65 Hz, 1H), 8.26 (d, *J*=8.41 Hz, 1H) ppm. ^13^C NMR (100 MHz, MeOD) δ=19.8, 21.5, 29.3, 46.7, 123.9, 124.4, 126.6, 126.8, 126.9, 127.5, 129.3, 129.3, 130.1, 132.5, 132.6, 135.7, 138.4, 139.2, 140.2, 142.1, 170.5 ppm. MS (ESI): m/z calcd for C_21_H_22_N_2_O_3_S [M+H]^+^383.15, found 383.2. Purity (LC, 254 nm): 93.6 %.

**2‐Methyl‐*N*‐(1‐phenyl)benzamide (4 a)**: 2‐Methylbenzoyl chloride (1 mL, 1.2 g, 8 mmol, 1.5 eq.) was added dropwise to a solution of TEA (1.5 mL, 1.1 g, 11 mmol, 2 eq.) and diethyl ether (5 mL). Next, 1‐phenylethanamine (0.7 mL, 0.67 g, 5.5 mmol, 1 eq.) was added slowly. After 1 h, the reaction mixture was diluted with ethyl acetate and washed with 0.1 M HCl (3x 10 mL), 5 % sodium carbonate solution (3x 10 mL) and saturated sodium chloride solution. The organic phase was dried over sodium sulfate and the solvent was evaporated. The crude product was recrystallized from methanol and water to yield **4 a** (480 mg, 2.0 mmol, yield: 36 %). mp: 110 °C. ^1^H NMR (400 MHz, CDCl_3_) δ=1.59 (d, *J*=6.82 Hz, 3H), 2.42 (s, 3H), 5.26–5.38 (m, 1H), 6.13 (d, *J*=7.07 Hz, 1H), 7.14–7.24 (m, 2H), 7.26–7.42 (m, 7H) ppm. ^13^C NMR (100 MHz, CDCl_3_) δ=19.7, 21.7, 49.0, 125.6, 126.1, 126.6, 127.3, 128.7, 129.7, 130.9, 136.0, 136.4, 143.1, 169.1 ppm. MS (ESI): m/z calcd for C_16_H_17_NO [M+H]^+^240.14, found 240.3. Purity (LC, 215 nm):>99 %.

**2‐Methyl‐*N*‐(naphth‐1‐ylethyl]benzamide (4 b)**: This compound was synthesized according to the general procedure Ac. 863 mg, 3.13 mmol, yield: 98 %. mp: 134 °C. ^1^H NMR (400 MHz, CDCl_3_) δ=2.48 (s, 3H), 5.10 (d, *J*=5.31 Hz, 2H), 5.98 (s, 1H), 7.14 (td, *J*=7.58, 0.51 Hz, 1H), 7.20 (d, *J*=7.58 Hz, 1H), 7.28 (td, *J*=7.58, 1.26 Hz, 2H), 7.32 (dd, *J*=7.58, 1.01 Hz, 1H), 7.39–7.49 (m, 1H), 7.49–7.64 (m, 3H), 7.85 (d, *J*=8.08 Hz, 1H), 7.87–7.96 (m, 1H), 8.15 (d, *J*=8.59 Hz, 1H) ppm. ^13^C NMR (100 MHz, CDCl_3_) δ=19.8, 42.2, 123.6, 125.4, 125.7, 126.1, 126.6, 126.7, 127.0, 128.8, 129.9, 131.0, 131.4, 133.5, 134.0, 136.2, 136.2, 169.6 ppm. MS (ESI): *m/z* calcd for C_19_H_17_NO [M+H]^+^276.13, found 276.2. Purity (LC, 300 nm):>99 %.

***N*** **‐(Diphenylmethyl)‐2‐methylbenzamide (4 c)**: 1,1‐diphenylmethanamine (53.7 μL, 57.2 mg, 0.312 mmol, 1 eq.), TEA (100 μL, 78 mg, 0.77 mmol, 2.5 eq.) and 2‐methylbenzoyl chloride (50 μL, 60 mg, 0.39 mmol, 1.25 eq.) were added to dichloromethane (10 mL). The reaction was monitored by TLC. After the reaction was completed, the mixture was washed with 1 M hydrochloric acid (3x 5 mL). The organic phase was dried, and the solvent was evaporated under reduced pressure. The crude product was purified by silica gel column chromatography to yield **4 c** (65 mg, 0.22 mmol, yield: 69 %). mp: 179 °C. R_f_=0.62 (CH/EtOAc 3 : 1). ^1^H NMR (400 MHz, CDCl_3_) δ=2.45 (s, 3H), 6.35 (d, *J*=7.78 Hz, 1H), 6.47 (d, *J*=8.03 Hz, 1H), 7.19–7.25 (m, 2H), 7.28–7.40 (m, 11H), 7.41–7.45 (m, 1H) ppm. ^13^C NMR (100 MHz, CDCl_3_) δ=19.9, 57.2, 125.8, 126.6, 127.4, 127.6, 128.7, 130.0, 131.1, 136.1, 136.4, 141.5, 169.0 ppm. MS (ESI): m/z calcd for C_21_H_19_NO [M+H]^+^302.16, found 302.2. Purity (LC, 215 nm): 93.9 %.

**2‐Methyl‐*N*‐[(1*S*)‐1‐naphth‐1‐ylethyl]benzamide (4 d)**: To a solution of (1*S*)‐1‐Naphth‐1‐ylethylamine (50 μL, 53.4 mg, 0.312 mmol, 1 eq.) and TEA (100 μL, 78 mg, 0.77 mmol, 2.5 eq.) in dichloromethane (2 mL), 2‐Methylbenzoyl chloride (50 μL, 60 mg, 0.39 mmol, 1.25 eq.) was added dropwise. After 1 h, the resulting precipitate was filtered off and washed with dichloromethane. The filtrate was freed from solvent and the residue was dissolved in ethyl acetate (10 mL). The solution was washed with 10 % citric acid solution (3x 5 mL) followed by saturated sodium chloride solution (5 mL). The organic phase was evaporated under reduced pressure and the crude product was recrystallized twice from ethyl acetate to yield 4d (61 mg, 0.21 mmol, yield: 68 %). mp: 160 °C. R_f_=0.48 (CH/EtOAc 3 : 1). ^1^H NMR (400 MHz, CDCl_3_) δ=1.81 (d, *J*=6.65 Hz, 3H), 2.45 (s, 3H), 5.99 (d, *J*=7.78 Hz, 1H), 6.09–6.24 (m, 1H), 7.09–7.22 (m, 2H), 7.24–7.31 (m, 2H), 7.43–7.65 (m, 4H), 7.83 (d, *J*=8.16 Hz, 1H), 7.86–7.95 (m, 1H), 8.26 (d, *J*=8.53 Hz, 1H) ppm. ^13^C NMR (100 MHz, CDCl_3_) δ=19.7, 20.6, 44.9, 122.6, 123.6, 125.2, 125.6, 126.0, 126.5, 126.6, 128.5, 128.8, 129.8, 130.9, 131.2, 134.0, 136.1, 136.4, 138.0, 168.9 ppm. MS (ESI): m/z calcd for C_20_H_19_NO [M+H]^+^290.16, found 290.2. Purity (LC, 254 nm): 98.6 %.

***N*** **‐(2‐Hydroxy‐1‐naphth‐1‐ylethyl)‐2‐methylbenzamide (8)**: S4 (107 mg, 0.25 mmol, 1 eq.) and lithium hydroxide monohydrate (210 mg, 5.0 mmol, 20 eq.) were dissolved in THF/H_2_O (3 : 1, 30 mL) and heated under reflux for 2 d. THF was evaporated under reduced pressure and residual aqueous solution was extracted with ethyl acetate (3x 10 mL) The combined organic extracts were dried over sodium sulfate and evaporated under reduced pressure. The crude product was purified by silica gel column chromatography to afford **8** (56 mg, 0.18 mmol, yield: 73 %) as an off‐white solid. mp: 97 °C. R_f_=0.31 (CH/EtOAc 1 : 1). ^1^H NMR (400 MHz, CDCl_3_) δ=2.45 (s, 3H), 2.98 (s., 1H), 4.05–4.21 (m, 2H), 6.08 (dt, *J=*7.28, 5.08 Hz, 1H), 6.61 (d, *J=*7.28 Hz, 1H), 7.11–7.26 (m, 2H), 7.28–7.61 (m, 6H), 7.82 (d, *J*=7.91 Hz, 1H), 7.86–7.93 (m, 1H), 8.16 (d, *J*=8.41 Hz, 1H) ppm. ^13^C NMR (100 MHz, CDCl_3_) δ=19.8, 52.0, 65.6, 122.9, 123.5, 125.1, 125.7, 126.0, 126.7, 126.7, 128.7, 129.0, 130.1, 130.9, 131.0, 134.1, 134.4, 135.9, 136.2, 170.5 ppm. ESI (MS) *m/z*: calcd for C_20_H_19_NO_2_ [M+H]^+^306.15, found 306.1. Purity (LC, 300 nm): 97.9 %.

***N*** **‐(2‐Methylphenyl)‐2‐naphth‐1‐ylpropanamide (11 a)**: This compound was synthesized according to the general procedure B. 52 mg, 0.18 mmol, yield: 45 %. mp: 151 °C. ^1^H NMR (400 MHz, CDCl_3_) δ=1.54 (s, 3H), 1.87 (d, *J*=7.33 Hz, 3H), 4.54 (q, *J*=7.33 Hz, 1H), 6.78 (s., 1H), 6.93–7.02 (m, 2H), 7.10–7.21 (m, 1H), 7.48–7.61 (m, 3H), 7.63 (d, *J*=6.82 Hz, 1H), 7.76–7.96 (m, 3H), 8.14 (d, *J*=8.08 Hz, 1H) ppm. ^13^C NMR (100 MHz, CDCl_3_) δ=16.7, 17.5, 44.7, 122.2, 123.3, 124.8, 125.0, 125.6, 126.1, 126.6, 126.9, 128.3, 128.5, 129.0, 130.2, 131.6, 134.1, 135.5, 136.4, 172.7 ppm. (ESI): *m/z* calcd for C_20_H_19_NO [M+H]^+^290.16, found 290.2. Purity (LC, 254 nm): 98.8 %.

***N*** **‐(2‐Fluorphenyl)‐2‐naphth‐1‐ylpropanamide (11 b)**: This compound was synthesized according to the general procedure B. 70 mg, 0.24 mmol, yield: 60 %. mp: 116 °C. ^1^H NMR (400 MHz, CDCl_3_) δ=1.81 (d, *J*=7.15 Hz, 3H), 4.54 (q, *J*=7.15 Hz, 1H), 6.90–7.01 (m, 2H), 7.09 (t, *J*=7.40 Hz, 1H), 7.26 (s., 1H), 7.48–7.63 (m, 4H), 7.86 (d, *J*=8.16 Hz, 1H), 7.89–7.94 (m, 1H), 8.12 (d, *J*=8.28 Hz, 1H), 8.29 (t, *J*=7.59 Hz, 1H) ppm. ^13^C NMR (100 MHz, CDCl_3_) δ=17.9, 44.8, 114.6, 121.6, 123.0, 124.2, 124.4, 125.0, 125.7, 126.1, 126.3, 126.9, 128.6, 129.2, 131.5, 134.2, 136.2, 172.8 ppm. MS (ESI): *m/z* calcd for C_19_H_16_FNO [M+H]^+^294.13, found 294.2. Purity (LC, 254 nm): 96.0 %.

**2‐Naphth‐1‐yl‐*N*‐(4‐sulfamoylphenyl)propanamide (11 c)**: This compound was synthesized according to the general procedure B. 27 mg, 0.08 mmol, yield: 19 %. mp: 206 °C. R_f_=0.8 (EtOAc). ^1^H NMR (400 MHz, acetone‐*d*_6_) δ=1.62 (d, *J*=7.07 Hz, 3H), 4.68 (q, *J*=6.82 Hz, 1H), 6.43 (s., 2H), 7.42–7.64 (m, 4H), 7.79 (s, 4H), 7.83 (d, *J*=8.08 Hz, 1H), 7.93–7.97 (m, 1H), 8.22 (d, *J*=8.59 Hz, 1H), 9.59 (s., 1H) ppm. ^13^C NMR (100 MHz, acetone‐*d*_6_) δ=19.2, 44.1, 119.7, 119.8, 124.3, 125.3, 126.6, 126.7, 127.2, 128.0, 128.5, 129.9, 132.3, 135.2, 139.0, 139.4, 143.6, 173.9 ppm. MS (ESI): *m/z* calcd for C_19_H_18_N_2_O_3_S [M−H]^−^ 353.09, found 353.2. Purity (LC, 254 nm): 98.6 %.

***N*** **‐Cyclohexyl‐2‐naphth‐1‐ylpropanamide (11 d)**: This compound was synthesized according to the general procedure B. 53 mg, 0.19 mmol, yield: 47 %. mp: 151 °C. R_f_=0.3 (CH/EtOAc 3 : 1). ^1^H NMR (400 MHz, CDCl_3_) δ=0.78–0.87 (m, 2H), 1.14–1.36 (m, 4H), 1.50 (m, 2H), 1.64–1.78 (m, 2H), 1.70 (d, *J*=7.15 Hz, 3H), 3.64–3.82 (m, 1H), 4.28 (q, *J*=7.19 Hz, 1H), 5.08 (s., 1 H), 7.40–7.58 (m, 4H), 7.78–7.83 (m, 1H), 7.86–7.92 (m, 1H), 8.01–8.07 (m, 1H) ppm. ^13^C NMR (100 MHz, acetone‐*d*_6_) δ=18.0, 24.6, 25.4, 32.7, 32.7, 43.9, 48.1, 123.4, 124.9, 125.6, 125.9, 126.5, 128.1, 129.0, 131.5, 134.0, 137.3, 173.7 ppm. MS (ESI): *m/z* calcd for C_19_H_23_NO [M+H]^+^282.19, found 282.3. Purity (LC, 254 nm): 94.8 %.

**4‐Methyl‐2‐[(1*R*)‐1‐naphth‐1‐ylethyl]‐2,3‐dihydro‐1*H*‐isoindol hydrochloride (14 a)**: This compound was synthesized according to the general procedure E. 84 mg, 0.17 mmol, yield: 23 % as off‐white solid. mp: 247 °C. R_f_=0.75 (CH/EtOAc 3 : 1). ^1^H NMR (400 MHz, MeOD) δ=1.94 (d, *J*=6.65 Hz, 3H 46 %), 1.97 (d, *J*=6.65 Hz, 3H 54 %), 2.03 (s, 3H 46 %), 2.39 (s, 3H 54 %), 4.24–4.42 (m, 2H), 4.90–5.26 (m, 2H), 5.82 (q, *J*=6.65 Hz, 1H), 6.91–7.37 (m, 3H), 7.56–7.74 (m, 3H), 7.96–8.10 (m, 3H), 8.32 (t, *J*=8.03 Hz, 1H) ppm. ^13^C NMR (100 MHz, MeOD) δ=18.6, 18.9, 20.4, 58.5, 58.7, 59.7, 59.8, 61.6, 121.3, 123.3, 126.2, 127.0, 127.8, 128.9, 130.5, 130.6, 131.1, 131.5, 132.3, 133.8, 134.1, 134.3, 134.4, 134.5, 134.8, 135.0, 135.8 ppm. MS (ESI): *m/z* calcd for C_21_H_22_ClN [M+H]^+^288.18, found 288.1. Purity (LC, 280 nm):>99 %.

**4‐Fluor‐2‐[(1*R*)‐1‐naphth‐1‐ylethyl]‐2,3‐dihydro‐1*H*‐isoindol chloride (14 b)**: This compound was synthesized according to the general procedure E. 46 mg, 0.14 mmol, yield: 8 % as colorless solid. mp: decomp.>260 °C. R_f_=0.66 (PE/EtOAc 6 : 1). ^1^H NMR (400 MHz, acetone‐*d*_6_ and D_2_O) δ=1.96 (d, *J*=6.78 Hz, 3H), 4.41 (s, 2H), 5.02 (d, *J*=11.80 Hz, 1H), 5.11–5.39 (m, 1H), 5.83 (q, *J*=6.73 Hz, 1H), 6.98–7.55 (m, 3H), 7.59–7.75 (m, 3H), 7.91 (d, *J*=7.40 Hz, 1H), 8.05 (dd, *J*=10.67, 8.53 Hz, 2H), 8.31 (d, *J*=8.53 Hz, 1H) ppm. ^13^C NMR (100 MHz, acetone‐*d*_6_ and D_2_O) δ=20.2, 55.7, 58.9, 61.1, 116.0, 119.8, 123.0, 126.0, 126.1, 126.8, 127.3, 128.3, 130.1, 130.8, 131.4, 132.3, 133.8, 134.8, 138.2 ppm. MS (ESI): *m/z* calcd for C_20_H_19_ClFN [M+H]^+^292.15, found 292.1. Purity (LC, 280 nm):>99 %.

**2‐(Naphth‐1‐ylmethyl)‐2,3‐dihydro‐1*H*‐isoindol hydrochloride (14 c)**: This compound was synthesized according to the general procedure D. 120 mg, 0.406 mmol, yield: 81 % as dark blue solid. mp: decomp.>150 °C. R_f_=0.59 (CH/EtOAc 3 : 1). ^1^H NMR (400 MHz, DMSO‐*d*_6_) δ=4.54–4.78 (m, 4H), 5.14 (d, *J*=5.52 Hz, 2H), 7.26–7.45 (m, 4H), 7.56–7.73 (m, 3H), 7.99–8.13 (m, 3H), 8.43 (d, *J*=8.28 Hz, 1H), 11.88 (s, 1H) ppm. ^13^C NMR (100 MHz, DMSO‐*d*_6_) δ=53.0, 57.7, 122.8, 123.8, 125.5, 126.3, 127.0, 127.3, 128.3, 128.8, 130.1, 130.2, 131.5, 133.4, 134.4 ppm. MS (ESI): *m/z* calcd for C_19_H_18_ClN [M+H]^+^260.15, found 260.1. Purity (LC, 280 nm): 86.9 %.

**5‐Methoxy‐2‐[(1*R*)‐1‐naphth‐1‐ylethyl]‐2,3‐dihydro‐1*H*‐isoindol (14 d)**: 1,2‐*Bis*(bromomethyl)‐4‐methoxybenzene **15 a** (343 mg, 1.17 mmol, 1 eq.) was dissolved in acetonitrile (25 mL) and (1*R*)‐1‐naphth‐1‐ylethylamine (187 μL, 200 mg, 1.17 mmol, 1 eq.) was added dropwise. The reaction was heated under reflux for 4 h. The solvent was removed under reduced pressure and the residue was taken up in ethyl acetate (20 mL). The solution was washed with 10 % sodium carbonate solution (3x). The organic phase was dried over sodium sulfate and the solvent was evaporated under reduced pressure. The crude product was purified by silica gel column chromatography (PE/EtOAc 5 : 1). 118 mg, 0.389 mmol, yield: 33 % as dark red viscous oil. R_f_=0.56 (CH/EtOAc 3 : 1). ^1^H NMR (400 MHz, CDCl_3_) δ=1.65 (d, *J*=6.53 Hz, 3H), 3.78 (s, 3H), 3.83–4.09 (m, 4H), 4.47 (q, *J*=5.94 Hz, 1H), 6.70–6.79 (m, 2H), 7.07 (d, *J*=8.16 Hz, 1H), 7.45–7.57 (m, 3H), 7.72–7.84 (m, 2H), 7.86–7.95 (m, 1H), 8.53 (d, *J*=4.39 Hz, 1H) ppm. MS (ESI): *m/z* calcd for C_21_H_21_NO [M+H]^+^304.17, found 304.1. Purity (LC, 280 nm):>99 %.

**2‐[(1*R*)‐1‐Naphth‐1‐ylethyl]‐4‐nitro‐2,3‐dihydro‐1*H*‐isoindole (14 e)**: This compound was synthesized according to the general procedure D. 301 mg, 0.945 mmol, yield: 64 % as red oil. R_f_=0.32 (PE/EtOAc 6 : 1). ^1^H NMR (400 MHz, DMSO‐*d*_6_) δ=1.68 (s., 3H), 4.01 (s., 2H), 4.40 (d, *J*=16.44 Hz, 1H), 4.47–4.58 (m, 1H), 4.63 (d, *J*=15.94 Hz, 1H), 7.33–7.59 (m, 5H), 7.66–7.75 (m, 1H), 7.81 (d, *J*=8.66 Hz, 1H), 7.88–7.95 (m, 1H), 8.05 (d, *J*=7.91 Hz, 1H), 8.50 (s., 1H) ppm. MS (ESI): *m/z* calcd for C_20_H_18_N_2_O_2_ [M+H]^+^319.15, found 319.1. Purity (LC, 280 nm):>99 %.

**2‐[(1*R*)‐1‐Naphth‐1‐ylethyl]‐5‐nitro‐2,3‐dihydro‐1*H*‐isoindole (14 f)**: This compound was synthesized according to the general procedure D. 100 mg, 0.31 mmol, yield: 36 % as yellow viscous oil. R_f_=0.40 (PE/EtOAc 6 : 1). ^1^H NMR (400 MHz, CDCl_3_) δ=1.67 (s, 3H), 3.93–4.17 (m, 4H), 4.49 (s, 1H), 7.29 (d, *J*=8.53 Hz, 1H), 7.46–7.57 (m, 3H), 7.65–7.77 (m, 1H), 7.82 (d, *J*=7.91 Hz, 1H), 7.86–7.97 (m, 1H), 8.02 (s, 1H), 8.10 (d, *J*=8.16 Hz, 1H), 8.49 (s, 1H) ppm. MS (ESI): *m/z* calcd. for C_20_H_18_N_2_O_2_ [M+H]^+^319.15, found 319.1. Purity (LC, 280 nm):>99 %.

**4‐Chlor‐2‐[(1*R*)‐1‐naphth‐1‐ylethyl]‐2,3‐dihydro‐1*H*‐isoindol (14 g)**: This compound was synthesized according to the general procedure D. 189 mg, 0.614 mmol, yield: 74 % as reddish oil. R_f_=0.52 (CH/EtOAc 6 : 1). ^1^H NMR (400 MHz, DMSO‐*d*_6_) δ=1.66 (d, *J*=6.65 Hz, 3H), 3.89–4.26 (m, 4H), 4.49 (q, *J*=6.06 Hz, 1H), 7.04 (d, *J*=7.03 Hz, 1H), 7.09–7.21 (m, 2H), 7.44–7.59 (m, 3H), 7.74 (d, *J*=7.03 Hz, 1H), 7.81 (d, *J*=8.16 Hz, 1H), 7.86–7.96 (m, 1H), 8.52 (d, *J*=6.40 Hz, 1H) ppm. ^13^C NMR (100 MHz, DMSO‐*d*_6_) δ=22.3, 57.2, 58.7, 62.1, 120.5, 123.7, 124.9, 125.4, 125.7, 125.8, 126.8, 127.6, 128.3, 128.7, 128.9, 131.0, 134.1, 138.6, 140.5, 142.2 ppm. MS (ESI): *m/z* calcd for C_20_H_18_ClN [M+H]^+^308.12, found 308.1. Purity (LC, 280 nm):>97.8 %.

**2‐[(1*R*)‐1‐Naphth‐1‐ylethyl]‐2,3‐dihydro‐1*H*‐isoindol hydrochloride (14 h)**: This compound was synthesized according to the general procedure D. 29 mg, 0.11 mmol, 89 % as colorless solid. mp: decomp.>240 °C. R_f_=0.33 (PE/EtOAc 6 : 1). ^1^H NMR (400 MHz, DMSO‐*d*_6_) δ=1.82 (d, *J*=6.53 Hz, 3H), 4.20 (ddd, *J*=33.63, 14.31, 7.53 Hz, 2H), 5.01 (ddd, *J*=60.92, 13.68, 6.21 Hz, 2H), 5.76–5.90 (m, 1H), 7.23 (d, *J*=7.40 Hz, 1H), 7.30 (t, *J*=7.40 Hz, 1H), 7.37 (t, *J*=7.40 Hz, 1H), 7.46 (d, *J*=7.65 Hz, 1H), 7.56–7.75 (m, 3H), 8.02–8.08 (m, 2H), 8.33 (d, *J*=7.15 Hz, 1H), 8.39 (d, *J*=8.41 Hz, 1H), 12.43 (s, 1H) ppm. ^13^C NMR (100 MHz, DMSO‐*d*_6_) δ=19.9, 57.3, 57.7, 59.2, 122.5, 122.7, 122.9, 125.5, 125.8, 126.2, 127.1, 128.2, 128.4, 129.0, 129.3, 130.4, 133.5, 134.0, 134.3, 134.6 ppm. MS (ESI): *m/z* calcd for C_20_H_20_ClN [M+H]^+^274.16, found 274.2. Purity (LC, 280 nm): 94.3 %.

**2‐[(1*S*)‐1‐Naphth‐1‐ylethyl]‐2,3‐dihydro‐1*H*‐isoindol hydrochloride (14 i)**: This compound was synthesized according to the general procedure D. 203 mg, 0.655 mmol, yield: 91 % as brown solid. mp: decomp.>236 °C. R_f_=0.66 (CH/EtOAc 3 : 1). ^1^H NMR (400 MHz, DMSO‐*d*_6_) δ=1.81 (d, *J*=6.65 Hz, 3H), 4.12–4.33 (m, 2H), 5.02 (ddd, *J*=56.62, 13.77, 6.27 Hz, 2H), 5.77–5.91 (m, 1H), 7.19–7.27 (m, 1H), 7.31 (t, *J*=7.34 Hz, 1H), 7.38 (t, *J*=7.53 Hz, 1H), 7.44–7.49 (m, *J*=7.40 Hz, 1H), 7.59–7.71 (m, 3H), 8.05 (d, *J*=8.16 Hz, 2H), 8.20–8.29 (m, 1H), 8.40 (d, *J*=8.41 Hz, 1H), 12.04 (s, 1H) ppm. ^13^C NMR (100 MHz, DMSO‐*d*_6_) δ=19.9, 57.3, 57.7, 59.2, 122.5, 122.7, 122.9, 125.5, 125.8, 126.2, 127.1, 128.2, 128.4, 129.0, 129.3, 130.4, 133.5, 134.0, 134.3, 134.6 ppm. MS (ESI): *m/z* calcd for C_20_H_20_ClN [M+H]^+^274.16, found 274.2. Purity (LC, 280 nm): 98.5 %.

**2‐[(1*R*)‐1‐Naphth‐1‐ylethyl]‐2,3‐dihydro‐1*H*‐isoindol‐4‐amine (14 j)**: This compound was synthesized according to the general procedure F. 124 mg, 0.43 mmol, yield: 45 % as brownish solid. mp: decomp.>103 °C. ^1^H NMR (400 MHz, CDCl_3_) δ=1.67 (d, *J*=6.53 Hz, 3H), 3.52 (s, 2H), 3.86 (s, 2H), 3.96 (d, *J*=12.80 Hz, 1H), 4.13 (d, *J*=13.18 Hz, 1H), 4.50 (q, *J*=6.53 Hz, 1H), 6.52 (dd, *J*=7.84, 0.56 Hz, 1H), 6.64 (d, *J*=7.28 Hz, 1H), 7.04 (t, *J*=7.65 Hz, 1H), 7.43–7.59 (m, 3H), 7.72–7.85 (m, 2H), 7.86–7.95 (m, 1H), 8.56 (d, *J*=5.52 Hz, 1H) ppm. ^13^C NMR (100 MHz, CDCl_3_) δ=22.4, 55.5, 58.5, 62.3, 112.6, 113.2, 123.8, 124.8, 125.1, 125.4, 125.7, 125.7, 127.5, 128.1, 128.8, 131.0, 134.0, 140.7, 140.9, 141.2 ppm. MS (ESI): *m/z* calcd for C_20_H_19_ClFN [M+H]^+^289.17, found 289.1. Purity (LC, 280 nm): 97.9 %.

**2‐[(1*R*)‐1‐Naphth‐1‐ylethyl]‐2,3‐dihydro‐1*H*‐isoindol‐5‐amine (14 k)**: This compound was synthesized according to the general procedure F. 76 mg, 0.26 mmol, yield: 86 % as bright brown solid. mp: decomp.>84 °C. ^1^H NMR (400 MHz, CDCl_3_) δ=1.64 (d, *J*=6.53 Hz, 3H), 3.58 (s, 2H), 3.78–4.04 (m, 4H), 4.38–4.53 (m, 1H), 6.51 (s., 1H), 6.53 (dd, *J*=7.91, 2.13 Hz, 1H), 6.95 (d, *J*=7.91 Hz, 1H), 7.45–7.55 (m, 3H), 7.71–7.84 (m, 2H), 7.86–7.94 (m, 1H), 8.53 (d, *J*=4.52 Hz, 1H) ppm. ^13^C NMR (100 MHz, CDCl_3_) δ=22.5, 57.6, 58.2, 62.3, 109.2, 113.8, 122.9, 123.7, 125.1, 125.3, 125.7, 125.7, 127.4, 128.8, 130.1, 131.1, 134.0, 140.7, 141.3, 145.4 ppm. MS (ESI): *m/z* calcd for C_20_H_20_N_2_ [M+H]^+^289.17, found 289.2. Purity (LC, 280 nm): 88.5 %.

**2‐[(1*R*)‐1‐Naphth‐1‐ylethyl]‐2,3‐dihydro‐1*H*‐isoindol‐5‐ol hydrochloride (14 l)**: 5‐Methoxy‐2‐[(1*R*)‐1‐naphth‐1‐ylethyl]‐2,3‐dihydro‐1*H*‐isoindole (65 mg, 0.21 mmol, 1 eq.) was dissolved in 33 % HBr in acetic acid (20 mL) and heated to reflux for 24 h. The reaction mixture was concentrated and neutralized with semi‐saturated sodium bicarbonate. Then it was extracted with ethyl acetate (50 mL) and the organic phase was washed with saturated sodium chloride solution (2x 10 mL). The organic phase was dried over sodium sulfate and the solvent was evaporated under reduced pressure. The crude product was purified by silica gel column chromatography. 41 mg, 0.12 mmol, yield: 57 % as colorless crystals. mp: decomp.>240 °C. R_f_=0.32 (CH/EtOAc). ^1^H NMR (400 MHz, MeOD) δ=1.91 (d, *J*=6.78 Hz, 3H), 4.20–4.78 (m, 4H), 5.72 (q, *J*=6.11 Hz, 1H), 6.74 (s, 1H), 6.78 (dd, *J*=8.28, 1.88 Hz, 1H), 7.02–7.21 (m, 1H), 7.58–7.73 (m, 3H), 7.84 (d, *J*=7.15 Hz, 1H), 8.03 (t, *J*=7.65 Hz, 2H), 8.30 (d, *J*=8.53 Hz, 1H) ppm. ^13^C NMR (100 MHz, MeOD) δ=20.3, 59.1, 59.4, 110.7, 117.4, 123.3, 125.0, 126.9, 127.9, 128.9, 130.7, 131.5, 132.3, 135.8, 160.0 ppm. MS (ESI): *m/z* calcd for C_20_H_20_ClNO [M+H]^+^290.16, found 290.1. Purity (LC, 280 nm): 96.8 %.

**(1*R*)‐*N*‐(2‐Methylbenzyl)‐1‐naphth‐1‐ylethylamine hydrochloride (17)**: 1‐(Bromomethyl)‐2‐methylbenzene (115.4 mg, 0.624 mmol, 1.1 eq.), (*R*)‐1‐(naphthalen‐1‐yl)ethanamine (96 mg, 0.56 mmol, 1 eq.) and potassium carbonate(0.5 g, 3.6 mmol, 5 eq.) were dissolved in toluene (5 mL) and heated 12 h under reflux. The solvent was evaporated, and the residue was taken up in ethyl acetate (15 mL). The solution was washed twice with 10 % sodium carbonate solution (15 mL) followed by saturated sodium chloride solution (15 mL). The organic phase was dried over sodium sulfate and the solvent was evaporated. The crude product was purified by silica gel column chromatography (petroleum ether/ethyl acetate 5 : 1). The resulting isoindoline was taken up in HCl in methanol (4 M, 1 mL) and the solvent was evaporated under reduced pressure. (132 mg, 0.423 mmol, yield: 76 % as colorless solid. mp: 279 °C with decomp. R_f_=0.5 (CH/EtOAc 3 : 1). ^1^H NMR (400 MHz, MeOD) δ=1.87 (d, *J*=6.78 Hz, 3H), 2.09 (s, 3H), 3.99 (d, *J*=13.43 Hz, 1H), 4.25 (d, *J*=13.30 Hz, 1H), 5.51 (q, *J*=6.78 Hz, 1H), 7.19–7.34 (m, 3H), 7.40 (dd, *J*=7.40, 1.38 Hz, 1H), 7.58–7.69 (m, 3H), 7.84 (dd, *J*=7.22, 0.82 Hz, 1H), 7.99–8.06 (m, 2H), 8.15 (d, *J*=8.53 Hz, 1H) ppm. ^13^C NMR (100 MHz, MeOD) δ=19.2, 20.4, 48.2, 54.5, 123.1, 125.3, 126.8, 127.8, 128.0, 128.8, 130.6, 130.9, 131.1, 131.4, 131.8, 132.3, 132.5, 134.2, 135.7, 139.0 ppm. MS (ESI): *m/z* calcd. for C_20_H_22_ClN [M+H]^+^276.18, found 276.2. Purity (LC, 254 nm):>99 %.

**Fluorescence based enzyme‐activity assay. (SARS‐CoV PL^pro^)** A fluorometric assay was performed on a Cary Eclipse fluorimeter (Agilent Technologies, Santa Clara, USA) in white 96 well plates at 25 °C in a final volume of 200 μL. Z‐Arg‐Leu‐Arg‐Gly‐Gly‐AMC acetate was used as substrate at a final concentration of 50 μM, cleaved AMC was exited at 360 nm and detected at 460 nm. Inhibitors were provided as 2 mM DMSO stocks and diluted into assay buffer (20 mM Tris buffer pH 7.5, 0.1 mM EDTA, 200 mM NaCl) including 10 μL enzyme solution to a final concentration of 100 μM. For negative control of enzyme activity, measurements with DMSO were performed. For the determination of IC_50_‐values, the change in fluorescence intensity over time was measured for multiple inhibitor concentrations in duplicates. The resulting graph was fitted nonlinearly using GraFit (Erithacus Software Ltd., UK). For SARS‐CoV‐2 PL^pro^, the general assay conditions were identical, but SARS‐CoV PL^pro^ was exchanged for SARS‐CoV‐2 PL^pro^ and the assay was performed in presence of 1 mM dithiothreitol. Measurements were performed using a Spark 10 M microplate reader (Tecan Trading AG, Switzerland). SARS‐CoV PL^pro^ was kindly provided by the group of Prof. C. Kisker (Rudolf‐Virchow Zentrum, University of Würzburg, Germany). In the SI a method for the recombinant expression is given. SARS‐CoV‐2 PL^pro^ was expressed according to the procedure described in the SI.

### Cell‐based antiviral activity and cytotoxicity assays

**Cells and viruses**. Vero E6 cells were grown in Dulbecco's modified Eagle medium (DMEM) supplemented with 10 % fetal bovine serum (FBS) and antibiotics (100 U/mL penicillin and 100 μg/mL streptomycin) at 37 °C and an atmosphere containing 5 % CO_2_. SARS‐CoV‐2 was kindly provided by Christian Drosten (Institute of Virology, Charité‐Universitätsmedizin, Berlin).

**Cell toxicity**. Cytotoxic concentrations 50 % (CC_50_) of the compounds used in this study were determined as described previously.^[25]^

**Antiviral activity**. To determine the effective concentration 50 % (EC_50_) of the compounds, Vero E6 cells were inoculated with SARS‐CoV‐2 at a multiplicity of infection (MOI) of 0.5 plaque‐forming units (pfu) per cell. After incubation for 1 h at 33 °C, the virus inoculum was replaced with fresh cell culture medium containing the test compound at the indicated concentration. Following incubation for another 23 h at 33 °C, the cell culture supernatants were collected and virus titers were determined by virus plaque assay. Briefly, cells were seeded in 24‐well plates and confluent monolayers were inoculated with 10‐fold dilutions of virus‐containing cell culture supernatant. After 1 h, the virus inoculum was replaced with fresh minimum essential medium (MEM, Gibco) containing 1.25 % Avicel RC‐581 (FMC Biopolymer). After 3 d, the supernatant was removed, and cells were washed with phosphate‐buffered saline (PBS). Then, the cells were fixed with freshly prepared 3.7 % paraformaldehyde (in PBS) for 24 h and stained afterward with 0.15 % crystal violet. To calculate EC_50_ values, the virus titer determined for virus‐infected cells treated with solvent only (DMSO at the appropriate concentration) was used for normalization of virus titers obtained for inhibitor‐treated cells. Data from three independent experiments were used to calculate EC_50_ values by nonlinear regression analysis using GraphPad Prism 5.0 (GraphPad Software).

**Molecular docking**. For molecular docking studies, crystal structures of SARS‐CoV PL^pro^ in complex with **2 b** and **20** were used (PDB‐IDs 3E9S and 4OW0, respectively).^13,14^ By now, crystal structures of SARS‐CoV‐2 PL^pro^ are also available.[^26^, ^27^] However, these apo‐structures present a different orientation of the loop spanning residues 266–271 (corresponding to 267–272 in SARS‐CoV PL^pro^) with Tyr268 (Tyr269) not closed over the S3/S4 pocket. As ligands reported previously rely on interactions with this residue,^[21]^ these novel SARS‐CoV‐2 PL^pro^ crystal structures are less suitable for naphthyethylamine‐based inhibitor docking studies than the surrogate structures used herein. To consider both benzamide and basic isoindoline scaffolds, two docking protocols were developed. For benzamidine‐based inhibitors, docking was performed with crystal structure PDB‐ID 3E9S, while structure ID 4OW0 was used for isoindolines. In the 3E9S‐model all residues within 6.5 Å around the reference ligand were defined as part of the binding site. Additionally, water molecules 372, 388 and 393 were selected. For 4OW0 the same size definition was applied and water molecules 1035, 1041 and 1076 were included as part of the binding site. Both setups were validated by redocking and binder/non‐binder discrimination studies (SI‐Figure 4) using LeadIT‐2.3.2.^[28]^ Besides known binders and non‐binders, the discrimination set was enriched by the addition of decoys with similar physicochemical, but different topological properties from the database of useful decoys‐enhanced (DUD‐E).^[29]^ For the benzamide‐3E9S‐model 20 inhibitors and 12 non‐binders from this an previous studies^[20]^ were filled up with 150 decoys generated for **2 b**, **2 o** and **2 t** (50 decoys for each). The 4OW0‐model included 37 binders and 6 non‐binders from the reported isoindoline‐series (**14 h**–**l**) and piperidine‐scaffolds reported by Baez‐Santos *et al.*.^[21]^ Additional 200 decoys were generated for **14 h**, **20** and the ligands from PL^pro^‐inhibitor complexes with PDB‐IDs 3MJ5^[22]^ and 4OVZ.^[21]^ All molecules were protonated and energy‐minimized using the Merck Molecular Forcefied (MMFF94x)^[30]^ within MOE2019.01^[31]^ before docking. Graphics were made with PyMOL.,^[32]^ the Table of Contents image with SeeSAR‐10.^[33]^

## Conflict of interest

The authors declare no conflict of interest.

## Supporting information

As a service to our authors and readers, this journal provides supporting information supplied by the authors. Such materials are peer reviewed and may be re‐organized for online delivery, but are not copy‐edited or typeset. Technical support issues arising from supporting information (other than missing files) should be addressed to the authors.

Supplementary
