# Supplementary material for: Structure‐Activity Relationships of Benzamides and Isoindolines Designed as SARS‐CoV Protease Inhibitors Effective against SARS‐CoV‐2
Source: ChemMedChem. 2020 Oct 16;16(2):340–54. doi: 10.1002/cmdc.202000548 (PMC7894572; doi:10.1002/cmdc.202000548)
Supplement: Supplementary file 1 — Supplementary [file CMDC-16-340-s001.pdf]

ChemMedChem

Supporting Information

Structure-Activity Relationships of Benzamides and Isoindolines Designed as SARS-CoV Protease Inhibitors Effective against SARS-CoV-2

Armin Welker, Christian Kersten, Christin Müller, Ramakanth Madhugiri, Collin Zimmer, Patrick Müller, Robert Zimmermann, Stefan Hammerschmidt, Hannah Maus, John Ziebuhr, Christoph Sotriffer, and Tanja Schirmeister*

Synthetic procedures

SA General procedure for the introduction of a Boc protection group

The amine (1.0 eq.) was dissolved in a dioxane/water mixture (2:1). Di-*tert*-butyl dicarbonate (1.1 eq.) was added under stirring. For amines with carboxylic acid function, NaOH (3.0 eq.) were added for neutralization. After 2 h the mixture was acidified with 10% citric acid solution and extracted with dichloromethane (3x 50 mL). The organic phase was washed with saturated sodium chloride solution and dried over sodium sulfate. The solvent was evaporated under reduced pressure.

SB General procedure for the bromination after Wohl-Ziegler

The dimethylbenzene (1.0 eq.) was dissolved in THF (30 mL). *N*-bromosuccinimide (2.0 eq.) was added. The suspension was heated under reflux while being irradiated with a light bulb. The progress of reaction was monitored by TLC. After about 4 h, the reaction mixture was washed three times with water followed by three times saturated sodium chloride solution. The organic phase was dried over sodium sulfate and the solvent was evaporated. The crude mixture was purified by silica gel column chromatography.

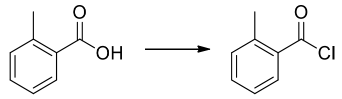

S1

SI-Scheme 1: Synthesis of of S1**.** Reagents and conditions: $SOCl_2$, DMF cat., 80 °C, 2 h, 95%.

2-Methylbenzoyl chloride (S1): 2-Methylbenzoic acid (20 g, 0.147 mol, 1.0 eq.) was suspended in thionyl chloride (30 mL, 49 g, 0.41 mL, 2.8 eq.) and 5 drops DMF were added. The mixture was heated under reflux under an argon atmosphere. After 2 h, the gas evolution was terminated, thionyl chloride was removed in vacuo and the crude product was used without purification.

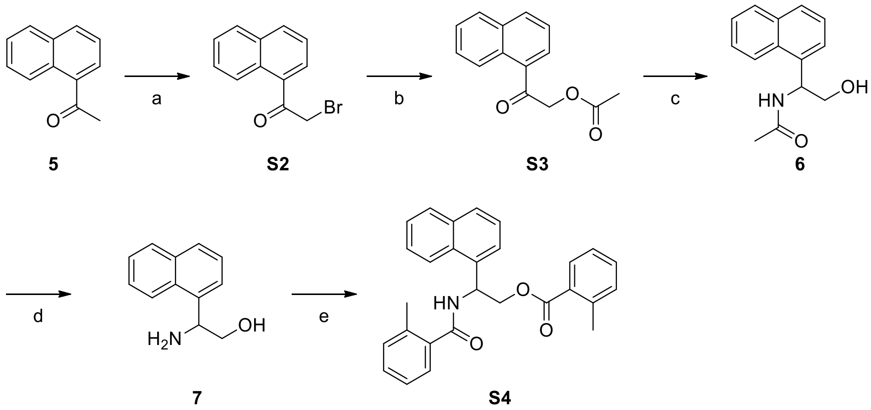

SI-Scheme 2: Synthesis of **S2, S3** and **S4**. Reagents and conditions: a) benzene, *N*-bromosuccinimide, *p*-toluoenesulfonic acid monohydrate, reflux, 16 h, 96%. b) H_2O, sodium acetate trihydrate, ethanol, reflux, 2 h, 98%. c) isopropanol, ammonia acetate, sodium cyanoborohydride, 95 °C, 1 h, 40%. d) semi-concentrated hydrochloric acid, reflux, 2 d. e) ethyl acetate, TEA, 2-methyl benzoyl chloride, r.t., 1 d, 90%.

2-Bromo-1-naphth-1-ylethanone (S2): A solution of 1-acetonaphthone (5.46 g, 32.1 mmol, 1 eq.), *N*-bromosuccinimide (6.28 g, 35.3 mmol, 1.1 eq.) and *p*-toluenesulfonic acid monohydrate (244 mg, 1.28 mmol, 4 mol%) in benzene (40 mL) was heated under reflux overnight. The mixture was diluted with ethyl acetate (30 mL) and the organic layer was extracted with 5% sodium carbonate solution (3x 50 mL) followed by saturated sodium chloride (50 mL). The organic layer was dried over magnesia sulfate and the solvent was

evaporated under reduced pressure to yield crude **S2** (7.67 g, 30.8 mmol, yield: 96%) as a dark oil which was used without further purification. R_f = 0.68 (CH/EtOAc 3:1). ^{1}H NMR (400 MHz, $CDCl_3$) δ = 4.58 (s, 2H), 7.52 (dd, *J* = 8.16, 7.28 Hz, 1H), 7.57(ddd, *J* = 8.03, 6.78, 1.25 Hz, 1H), 7.64 (ddd, *J* = 8.50, 6.93, 1.51 Hz, 1H), 7.88–7.92 (m, 1H), 7.93 (dd, *J* = 7.28, 1.13 Hz, 1H), 8.05 (d, *J* = 8.16 Hz, 1H), 8.61–8.67 (m, 1H) ppm. ^{13}C NMR (100 MHz, $CDCl_3$) δ = 33.8, 124.2, 125.7, 126.8, 128.5, 128.5, 130.6, 132.3, 133.9, 134.0, 194.3 ppm. MS (ESI): *m/z* calcd for $C_{12}H_9BrO$ $[M+H]^+$ 248.98, found 249.1.

2-Naphth-1-yl-2-oxoethylacetate (S3): Sodium acetate trihydrate (3.12 g, 22.9 mmol, 2.8 eq.) was dissolved in water (5 mL) and 2-bromo-1-naphth-1-ylethanone **S2** (2.21 g, 8.11 mmol, 1 eq.) and ethanol (30 mL) were added. The reaction mixture was heated under reflux for 2 h. The solvent was evaporated under reduced pressure, the residue was dissolved in ether (50 mL) and washed with water (2x 30 mL) followed by saturated sodium chloride (30 mL). The organic layer was dried over sodium sulfate and the solvent was evaporated under reduced pressure to afford crude **S3** (1.89 g, 7.95 mmol, yield: 98%) as a colorless oil which was used without further purification. R_f = 0.51 (CH/EtOAc 3:1). ^{1}H NMR (400 MHz, $CDCl_3$) δ = 2.26 (s, 3H), 5.32 (s, 2H), 7.52 (dd, *J* = 8.16, 7.28 Hz, 1H), 7.57 (ddd, *J* = 8.16, 6.78, 1.25 Hz, 1H), 7.62 (ddd, *J* = 8.28, 6.78, 1.51 Hz, 1H), 7.86 (dd, *J* = 7.22, 1.19 Hz, 1H), 7.88–7.92 (m, 1H), 8.05 (d, *J* = 8.28 Hz, 1H), 8.63 (d, *J* = 9.03 Hz, 1H) ppm.

***N*-(2-Hydroxy-1-naphth-1-ylethyl)acetamide (6):** Ammonia acetate (4.2 g, 55 mmol, 21 eq.), sodium cyanoborohydride (0.517 g, 8.23 mmol, 3.1 eq.) and 2-Naphth-1-yl-2-oxoethylacetate **S3** (0.01 g, 2.63 mmol, 1 eq.) were dissolved in isopropanol (20 mL) and heated to 95 °C for 1 h. The solvent was evaporated under reduced pressure and the residue was suspended in ethyl acetate. The suspension was filtered, and the filtrate diluted with petroleum ether (20 mL). The resulting precipitate was filtered off. The crude product was purified by silica gel column chromatography to give **6** (239 mg, 1.04 mmol, yield: 40%) as a colorless solid. ^{1}H NMR (400 MHz, DMSO-d_6) δ = 1.89 (s, 3H), 3.55–3.66 (m, 1H), 3.69–3.79 (m, 1H), 4.95 (t, *J* = 5.71 Hz, 1H), 5.67 (td, *J* = 7.81, 5.08 Hz, 1H), 7.44–7.62 (m, 4H), 7.82 (d, *J* = 7.91 Hz, 1H), 7.91–7.96 (m, 1H), 8.16 (d, *J* = 8.03 Hz, 1H), 8.40 (d, *J* = 8.28 Hz, 1H) ppm. ^{13}C NMR (100 MHz, DMSO-d_6) δ = 22.7, 51.0, 63.9, 123.02, 123.6, 125.3, 125.5, 126.1, 128.6, 168.8 ppm.

2-Amino-2-naphth-1-ylethanol (7): 6 was dissolved in semi-concentrated hydrochloric acid and heated under reflux for 2 d. The pH of the reaction mixture was adjusted to > 10 with KOH and then extracted with ethyl acetate (3x 20 mL). The organic phase was dried over sodium sulfate and the solvent was evaporated under reduced pressure to afford **7** (52 mg, 0.28 mmol, yield: 43%) as slightly yellow solid. ^{1}H NMR (400 MHz, $CDCl_3$) δ = 2.59 (s., 3H), 3.66 (dd, *J* = 10.85, 8.09 Hz, 1H), 3.93 (dd, *J* = 10.92, 3.64 Hz, 1H), 4.89 (dd, *J* = 7.47, 3.20 Hz, 1H), 7.42–7.55 (m, 3H), 7.55–7.62 (m, 1H), 7.73–7.80 (m, 1H), 7.85–7.91 (m, 1H), 8.10 (d, *J* = 7.91 Hz, 1H) ppm. ^{13}C NMR (100 MHz, $CDCl_3$) δ = 52.6, 67.2, 122.5, 122.8, 125.4, 125.5, 126.1, 127.7, 128.9, 130.8, 133.7, 138.2 ppm.

2-(2-Methylbenzamido)-2-(naphthalen-1-yl)ethyl 2-methylbenzoate (S4): 7 (52 mg, 0.28 mmol, 1 eq.), TEA (200 µL, 1.54 mmol, 5.5 eq.) and 2-methylbenzoic chloride (100 µL, 0.78 mmol, 2.8 eq.). The reaction mixture was stirred at r.t. for 1 d and then diluted with ethyl acetate (10 mL). The solution was washed with 10% sodium carbonate solution (3x 10 mL) and the organic phase was dried over sodium sulfate. The solvent was evaporated under reduced pressure to give crude **S4** (107 mg, 0.25 mmol, yield: 90%) as a brown solid which was used without further purification. R_f = 0.51 (CH/EtOAc 3:1). ^{1}H NMR (400 MHz, $CDCl_3$) δ = 2.37 (s, 3H), 2.57 (s, 3H), 4.82–4.95 (m, 2H), 6.44 (d, *J* = 8.03, 1H), 6.48–6.56 (m, 1H), 7.12–7.35 (m, 6H), 7.41 (td, *J* = 7.53, 1.52 Hz, 1H), 7.46–7.68 (m, 4H), 7.87 (d, *J* = 8.28 Hz, 1H), 7.89–7.95 (m, 2H), 8.35 (d, *J* = 8.41 Hz, 1H) ppm. ESI (MS) *m/z*: calcd for $C_{28}H_{25}NO_3$ $[M-H]^-$ 422.17, found 422.3.

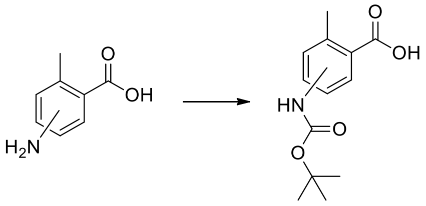

SI-Scheme 3: Synthesis of **S5a** and **b**. Reagents and conditions: Boc_2O, NaOH, dioxane, H_2O, 2 h, r.t. 57–73%.

4-[(*tert*-Butoxycarbonyl)amino]-2-methylbenzoic acid (S5a): This compound was synthesized according to the general procedure SA. 602 mg, 2.40 mmol, yield: 73%. ^{1}H NMR (400 MHz, $CDCl_3$) δ = 1.50 (s, 9H), 2.65 (s, 3H), 6.67 (s., 1H), 7.24–7.27 (m, 1H), 7.32 (d, *J* = 1.52 Hz, 1H), 8.04 (d, *J* = 8.59 Hz, 1H), 11.37 (s., 1H) ppm.

5-[(*tert*-Butoxycarbonyl)amino]-2-methylbenzoic acid (S5b): This compound was synthesized according to the general procedure SA. 236 mg, 0.94 mmol, yield: 57%. ^{1}H NMR (400 MHz, $CDCl_3$) δ = 1.52 (s, 9H), 2.50 (s, 3H), 7.16 (d, *J* = 8.34 Hz, 1H), 7.47 (dd, *J* = 8.34, 1.77 Hz, 1H), 7.94 (d, *J* = 2.27 Hz, 1H) ppm.

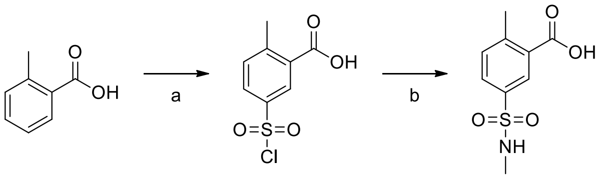

SI-Scheme 4: Synthesis of inhibitor **S7**. Reagents and conditions: a) HSO_3Cl, 0 °C–100 °C, 4 h, 43%. b) EtOAc, methylamine, r.t., 16 h, 95%.

5-Chlorosulfonyl-2-methylbenzoic acid (S6): Chlorosulfonic acid (6 mL, 10.5 g, 90 mmol, 4 eq.) was added slowly under argon atmosphere to 2-Methylbenzoic acid (3.0 g, 22 mmol, 1 eq.) and cooled to 0 °C. After the gas development was finished, the reaction mixture was warmed to r.t. and then heated to 100 °C for 4 h. The warm reaction mixture was poured in an ice-water mixture (200 mL). The resulting precipitate was filtered off, washed with ice cold water and dried to give crude **S6** which was used without further purification (2.23 g, 9.5 mmol, yield: 43%).

2-Methyl-5-(methylsulfamoyl)benzoic acid (S7): 5-Chlorosulfonyl-2-methylbenzoic acid (300 mg, 1.28 mmol, 1 eq.) was dissolved in ethyl acetate (2 mL) and 40% methylamine solution (0.2 g, 2.6 mmol, 2 eq.) was added dropwise. The reaction mixture was stirred overnight at r.t. and then acidified with 40% formic acid (1 mL). The resulting solution was extracted with dichloromethane (3x 5 mL). The organic phase was dried over sodium sulfate and the solvent was evaporated under reduced pressure to yield **S7** (278 mg, 1.21 mmol, yield: 95%). ^{1}H NMR (400 MHz, MeOD) δ = 2.53 (s, 3H), 2.67 (s, 3H), 7.50 (d, *J* = 8.16 Hz, 1H), 7.85 (dd, *J* = 8.03, 2.01 Hz, 1H), 8.34 (d, *J* = 2.01 Hz, 1H) ppm. ^{13}C NMR (100 MHz, MeOD) δ = 22.0, 29.3, 130.6, 131.1, 132.7, 133.8, 138.6, 146.4, 169.6 ppm.

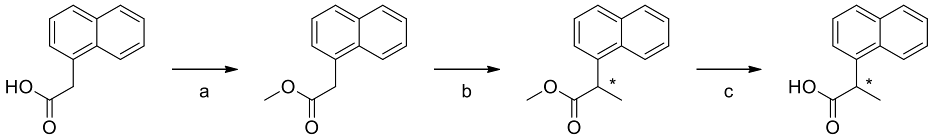

9 **S8** **S9** **10**

SI-Scheme 5: Synthesis of **10**. Reagents and conditions: a) MeOH, H_2SO_4, r.t.–65 °C, 2 h, 95%. b) MeI, DMSO, KOH, r.t., 10 d, 31%. c) LiOH, THF, H_2O, r.t., 16 h, 99%.

Methyl-naphth-1-ylacetate (S8): Naphthylacetic acid (4.3 g, 23.1 mmol, 1 eq.) were dissolved in Methanol (50 mL) and sulfuric acid (0.8 mL) was added. The reaction mixture was heated under reflux for 2 h and then cooled to r.t.. The pH value was adjusted to 5 with saturated sodium bicarbonate solution and the solution was concentrated on the rotary evaporator. Extraction with dichloromethane (3x 10 mL) provided the crude product, which was purified by silica gel column chromatography (gradient pentane/EtOAc 20:1–3:1) to yield **S8** (4.39 g, 21.9 mmol, yield: 95%). R_f = 0.7 (CH/EtOAc 1:1). ^{1}H NMR (400 MHz, $CDCl_3$) δ = 3.70 (s, 3H), 4.10 (s, 2H), 7.40–7.59 (m, 4H), 7.81 (dd, *J* = 7.47, 1.82 Hz, 1H), 7.85–7.91 (m, 1H), 7.98–8.03 (m, 1H) ppm. ^{13}C NMR (100 MHz, $CDCl_3$) δ = 39.0, 52.1, 123.7, 125.5, 125.8, 126.4, 128.0, 128.1, 128.7, 130.5, 132.1, 133.8, 172.0 ppm.

Methyl-2-naphth-1-ylpropanoate (S9): Naphthylacetic acid methyl ester **S8** (4.05 g, 20.2 mmol, 1 eq.), Methyl iodide (4.88 g, 34.4 mmol, 1.7 eq.) and KOH (1.23 g, 22 mmol, 1.1 eq.) were dissolved in DMSO (20 mL). The mixture was stirred at r.t. for 10 d and then diluted in a solution from sodium dihydrogen phosphate (5.0 g, 36 mmol, 1.8 eq.) in water (100 mL). The pH value of the solution was adjusted to 7 with saturated sodium bicarbonate solution. It was extracted with dichloromethane (4x 40 mL) and ethyl acetate (2x 40 mL) and the combined organic extracts were dried over sodium sulfate. The solvent was evaporated under reduced pressure to give the crude product **33S9**, which was purified by silica gel column chromatography (gradient: pentane/ETOAc 14:1–9:1). 1.327 g, 6.19 mmol, yield: 31%. R_f = 0.75 (CH/EtOAc 1:1). ^{1}H NMR (400 MHz, $CDCl_3$) δ = 400.13 MHz, $CDCl_3$: δ = 1.68 (d, *J* = 7.16 Hz, 3H), 3.67 (s, 3H), 4.53 (q, *J* =7.16 Hz, 1H), 7.45–7.59 (m, 4H), 7.77–7.82 (m, 1H), 7.86–7.92 (m, 1H), 8.10 (d, *J* = 8.34 Hz, 1H) ppm.

2-Naphth-1-ylpropanoic acid (10): Methyl-2-naphth-1-ylpropanoate **S9** (1.0 g, 4.7 mmol, 1 eq.) and lithium hydroxide monohydrate (0.294 g, 7 mmol, 1.5 eq.) were dissolved in a mixture of THF and water (2:1, 15 mL) and stirred at r.t. overnight. The reaction mixture was acidified with 1 M hydrochloric acid to pH 1 and then extracted with dichloromethane (3x 20 mL). Combined organic phases were dried over sodium sulfate and the solvent was evaporated under reduced pressure. 0.93 g, 4.6 mmol, yield: 99%. R_f = 0.2 (CH/EtOAc 3:1). ^{1}H NMR (400 MHz, DMSO-d_6) δ = 1.51 (d, *J* = 7.15 Hz, 3H), 4.47 (q, *J* = 7.03 Hz, 1H), 7.39–7.62 (m, 4H), 7.83 (d, *J* = 8.03 Hz, 1H), 7.91–7.99 (m, 1H), 8.13 (d, *J* = 8.41 Hz, 1H), 12.36 (s, 1H) ppm.

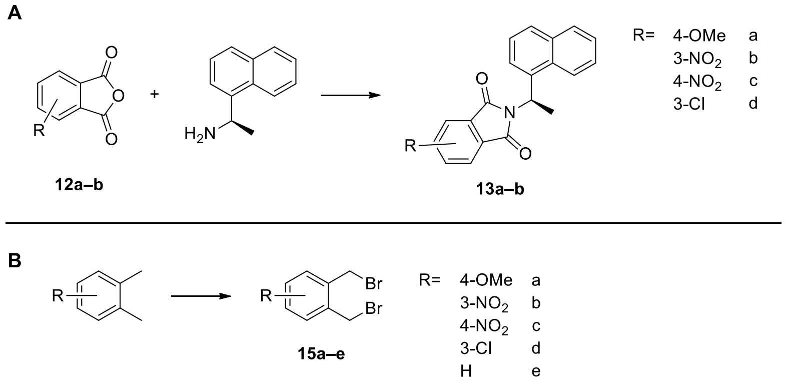

SI-Scheme 6: Synthesis of isoindoline derivatives. Reagents and conditions: **(A)**: heating with a heat gun, 27–92%. **(B)**: CCl_4, *N*-bromosuccinimide, 80 °C, h•v, 2 h, 6–89%.

4-Methyl-2-[(1*R*)-1-naphth-ylethyl]-1*H*-isoindol-1,3(2*H*)-dione (13a): 3-Methylphthalic acid anhydride (130 mg, 0.80 mmol, 1 eq.) and (1*R*)-1-Naphth-1-ylethylamine (136 mg, 0.79 mmol, 1 eq.) were melted with a heat gun. After cooling to r.t., the melt was taken up in acetic acid (2 mL) and the acetic acid was removed by heat gun. The procedure was repeated three times. The residue was then taken up in ethyl acetate and washed with water (10 mL) followed by saturated sodium chloride solution. The solvent was removed under reduced pressure and did not need any further purification. 230 mg, 0.73 mmol, yield: 92% as amorphous solid. ^{1}H NMR (400 MHz, $CDCl_3$) δ = 2.09 (d, *J* = 7.15 Hz, 3H), 2.64 (s, 3H), 6.38 (q, *J* = 7.07 Hz, 1H), 7.31 (d, *J* = 7.65 Hz, 1H), 7.40–7.53 (m, 2H), 7.55–7.65 (m, 3H), 7.86 (t, *J* = 8.72 Hz, 2H), 8.08 (d, *J* = 7.15 Hz, 1H), 8.30 (d, *J* = 8.53 Hz, 1H) ppm. MS (ESI): calcd for $C_{21}H_{17}NO_2$ $[M+H]^+$ 316.14, found 316.1.

4-Fluor-2-[(1*R*)-1-napht-1-ylethyl]-1*H*-isoindol-1,3(2*H*)-dione (13b): (1*R*)-1-NAphth-1-ylethanamine (331 mg, 1.93 mmol, 1 eq.) was dissolved in dichloromethane (5 mL) and 3-fluorphthalic acid anhydride (321 mg, 1.93 mmol, 1 eq.). The solvent was evaporated by a heat gun and the resulting residue was dissolved in acetic acid (5 mL). Xylol (5 mL) was added and evaporated again. 688 mg, 1.76 mmol, yield: 91% as a colorless oil. R_f = 0.33 (PE/EtOAc 6:1). ^{1}H NMR (400 MHz, $CDCl_3$) δ = 2.04 (d, *J* = 7.15 Hz, 3H), 6.33 (q, *J* = 7.15 Hz, 1H), 6.96–7.03 (m, 1H), 7.09–7.23 (m, 1H), 7.32 (t, *J* = 8.47 Hz, 1H), 7.44–7.61 (m, 3H), 7.81–7.89 (m, 2H), 8.02 (d, *J* = 7.15 Hz, 1H), 8.20 (d, *J* = 8.53 Hz, 1H) ppm.

1,2-Bis(bromomethyl)-4-methoxybenzene (15a)[1]: This compound was synthesized according to the general procedure SB. 0.80 g, 2.7 mmol, yield: 39% as a red viscous oil. R_f = 0.58 (CH/EtOAc 6:1). ^{1}H NMR (400 MHz, $CDCl_3$) δ = 3.83 (s, 3H), 4.63 (s, 2H), 4.67 (s, 2H), 6.84 (dd, *J* = 8.47, 2.70 Hz, 1H), 6.91 (d, *J* = 2.64 Hz, 1H), 7.30 (d, *J* = 8.41 Hz, 1H) ppm.

1,2-Bis(bromomethyl)-3-nitrobenzene (15b)[2]: This compound was synthesized according to the general procedure SB. 400 mg, 1.29 mmol, yield: 6% as a colorless oil. R_f = 0.44 (PE/EtOAc 6:1). ^{1}H NMR (400 MHz, $CDCl_3$) δ = 4.70 (s, 2H), 4.87 (s, 2H), 7.48 (t, *J* = 7.97 Hz, 1H), 7.65 (dd, *J* = 7.72, 1.32 Hz, 1H), 7.89 (dd, *J* = 8.16, 1.25 Hz, 1H) ppm.

1,2-Bis(bromomethyl)-4-nitrobenzene (15c)[3]: This compound was synthesized according to the general procedure SB. 375 mg crude product after silica gel column chromatography. The crude product was used in the next step without further purification. R_f = 0.64 (PE/EtOAc 6:1). ^{1}H NMR (400 MHz, $CDCl_3$) δ = 4.67 (s, 2H), 4.68 (s, 2H), 7.57 (d, *J* = 8.41 Hz, 1H), 8.15–8.19 (m, 1H), 8.26 (d, *J* = 2.38 Hz, 1H) ppm.

1,2-Bis(bromomethyl)-3-chlorbenzol (15d)[4]: This compound was synthesized according to the general procedure SB. 9.04 mg, 30.3 mmol, yield: 89% as reddish oil. R_f = 0.50 (CH). ^{1}H NMR (400 MHz, $CDCl_3$) δ = 4.63 (s, 2H), 4.82 (s, 2H), 7.22–7.27 (m, 1H), 7.28–7.32 (m, 1H), 7.39 (dd, *J* = 7.65, 1.63 Hz, 1H) ppm.

Antiviral activity and cytotoxicity

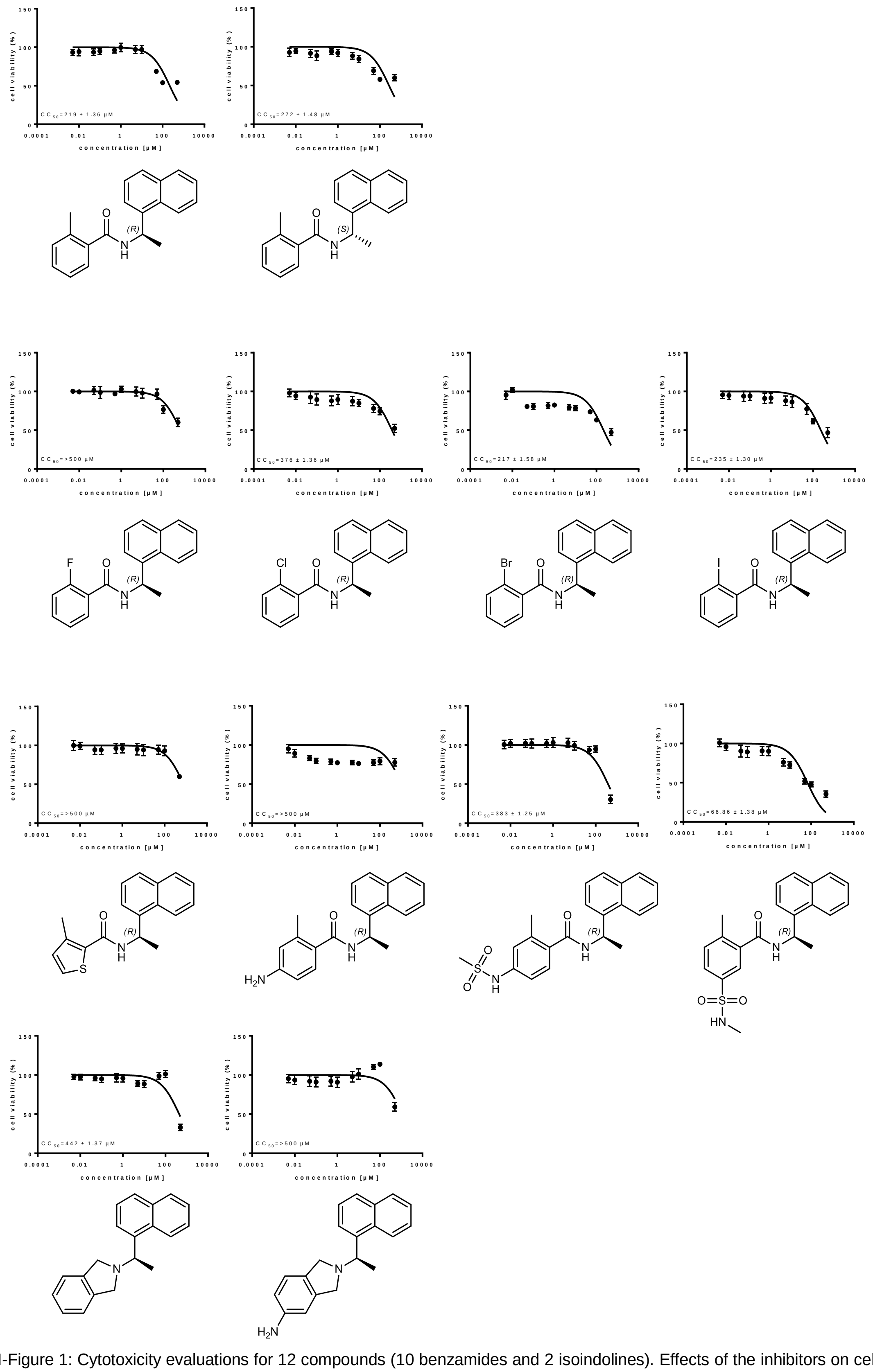

SI-Figure 1: Cytotoxicity evaluations for 12 compounds (10 benzamides and 2 isoindolines). Effects of the inhibitors on cell viability were determined by MTT assay using non-infected Vero E6 cells, which were incubated with serial dilutions of the respective inhibitor (solved in DMSO) for 24 h. Following the addition of MTT, the residual metabolic activity of cells was analyzed photometrically. The results were normalized using the values obtained for the corresponding DMSO concentration (non-linear regression analysis, GraphPad Prism 5.0). Experiments were performed in triplicate and means ± SEM are shown.

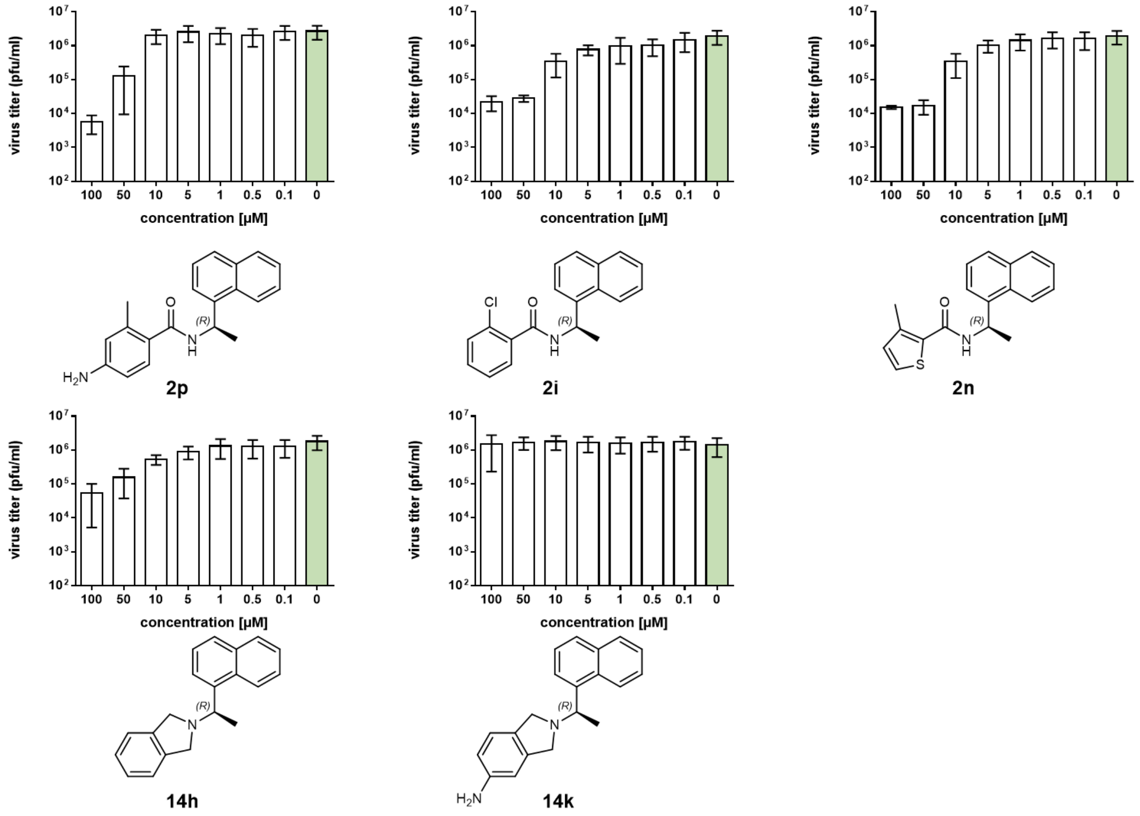

SI-Figure 2: Antiviral activity of different benzamide and isoindoline derivatives. Shown are virus titers (mean ± SEM) in the cell culture supernatants of Vero E6 cells infected with SARS-CoV-2 (MOI 0.5, 24 h post infection). Experiments were performed in triplicate and means ± SEM are shown. The green column indicates the virus titer in a control experiment (no inhibitor) in which virus-infected cells were treated with DMSO only.

Protein purification of SARS CoV PL^{pro}

The pET11-PLpro plasmid coding for the papain-like protease of SARS CoV and a poly-histidine tag for affinity chromatography was kindly provided by the group of Prof. C. Kisker (Rudolf-Virchow-Zentrum, University of Würzburg, Germany). Expression was performed as described previously by Baez-Santos *et al.*[5], with additional purification. Competent *Escherichia coli* Rosetta^{TM} 2 (DE3) pLysS cells (Merck, Darmstadt, Germany) were transformed with plasmid and grown in LB medium to an optical density of 0.8 at 37 °C. Overexpression of protein was induced by addition of IPTG in a final concentration of 500 µM and conducted at 37 °C for 4 hours. Cells were harvested by centrifugation, suspended in in lysis buffer$_{\text{SARS-CoV}}$ (20 mM Tris-HCl pH 7.4, 500 mM NaCl, 10 mM imidazole, 0.01 % Triton X-100), incubated with lysozyme (Carl Roth, Karlsruhe, Germany) for 60 minutes on ice and disrupted by sonication. Cell debris was removed by centrifugation to obtain a clear supernatant. Protein was purified from supernatant by affinity chromatography using a HisTrap HP column (GE Healthcare, Chicago, USA), which was first stripped and recharged with cobalt chloride. Histidine rich proteins and other impurities were removed by increasing stepwise the amount of elution buffer$_{\text{SARS-CoV}}$ (20 mM Tris-HCl pH 7.4, 500 mM NaCl, 500 mM imidazole), protein was finally eluted with 100% elution buffer. Further purification was conducted by size-exclusion chromatography (SEC) using a HiLoad 16/600 Superdex 75 pg (GE Healthcare, Chicago, USA) equilibrated in SEC buffer$_{\text{SARS-CoV}}$ (20 mM Tris-HCl pH 7.4, 150 mM NaCl, 10 mM DTT). For storage at –20 °C, protein was concentrated using Vivaspin-20 centrifugal filters with a 10 kDa cut-off (Sartorius, Göttingen, Germany), for cryo-protection, protein was diluted 1:1 with storage buffer$_{\text{SARS-CoV}}$ (20 mM Tris-HCl, 150 mM NaCl, 40 % glycerol), before flash freezing the protein in liquid nitrogen.

Plasmid construction of SARS CoV-2 PL^{pro}

The papain-like protease domain sequence was obtained from the solved crystal structure deposited in the Protein Data Bank by Osipiuk *et al.*[6] [PDB: 6W9C]. The sequence was codon optimized, synthesized and subcloned into pET28b vector between the NcoI and XhoI cleavage sites to receive a His6-tag by Genescript Biotech (Leiden, Netherlands). Sequence was verified by forward and reverse sequencing using the standard T7 and T7term primers (Eurofins Genomics Germany GmbH, Ebersberg, Germany).

Protein purification of SARS CoV-2 PL^{pro}

Purification was established based on Shin *et al.*[7] with some adjustments. In brief, competent *Escherichia coli* BL21-Gold (DE3) cells (Agilent Technologies, Santa Clara, USA) were transformed with plasmid and grown in LB medium to an optical density of 0.75 at 37 °C. Protein expression was induced by addition of IPTG to a final concentration of 500 µM, zinc chloride was added to a final concentration of 1 mM. Protein expression was conducted overnight at 18 °C. Cells were harvested by centrifugation, resuspended in lysis buffer$_{SARS\text{-}CoV\text{-}2}$ (50 mM Tris-HCl pH 7.5, 150 mM NaCl, 10 mM imidazole, 2 mM DTT, 0.1% Tween 20), incubated with lysozyme (Carl Roth, Karlsruhe, Germany) on ice for 30 min and lysed by sonication. Cell debris was removed by centrifugation to obtain a clear supernatant. Protein was purified from supernatant by affinity chromatography using a HisTrap HP column (GE Healthcare, Chicago, USA). Histidine-rich proteins and other impurities were removed by increasing stepwise the amount of elution buffer$_{SARS\text{-}CoV\text{-}2}$ (50 mM Tris-HCl pH 7.5, 150 mM NaCl, 250 mM imidazole, 2 mM DTT, 0.1% Tween 20), protein was finally eluted with 100% elution buffer. Further purification was conducted by SEC using a HiLoad 16/600 Superdex 75 pg (GE Healthcare, Chicago, USA) equilibrated in SEC buffer$_{SARS\text{-}CoV\text{-}2}$ (50 mM Tris-HCl pH 7.5, 150 mM NaCl, 2 mM DTT, 0.1% Tween 20). For storage at –20 °C, protein was concentrated using Vivaspin-20 centrifugal filters with a 10 kDa cut-off (Sartorius, Göttingen, Germany), for cryo-protection, 99% glycerol was added very gently to a final amount of 20%, before flash freezing the protein in liquid nitrogen.

Computational evaluations

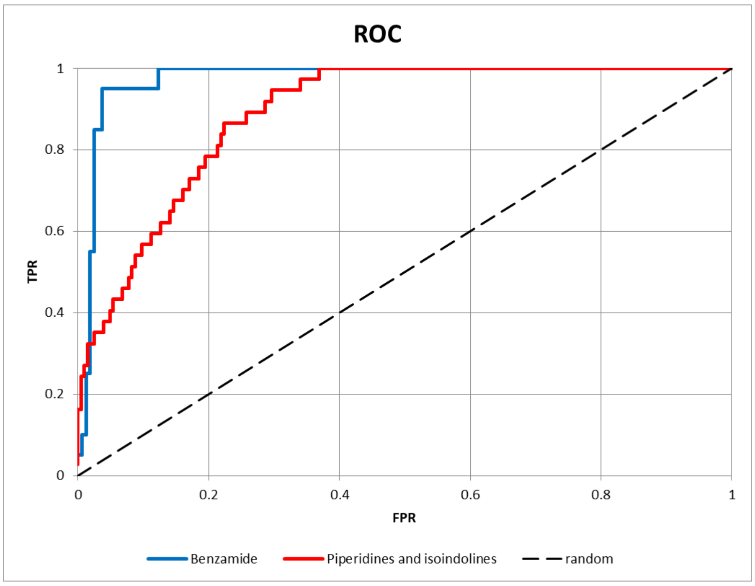

SI-Figure 3: Receiver operator characteristic (ROC) curves for benzamide-inhibitors (blue, PDB-ID 3E9S, redocking RMSD = 0.4 Å, 20 binder, 12 known non-binder, 150 decoys, AUC = 0.99) and piperidine/isoindoline (red, PDB-ID 4OW0, redocking RMSD = 1.6 Å, 37 binder, 6 known non-binder, 200 decoys, AUC = 0.89) molecular docking model. TPR: true positive rate, FPR: false positive rate. The dotted line displays random distribution.

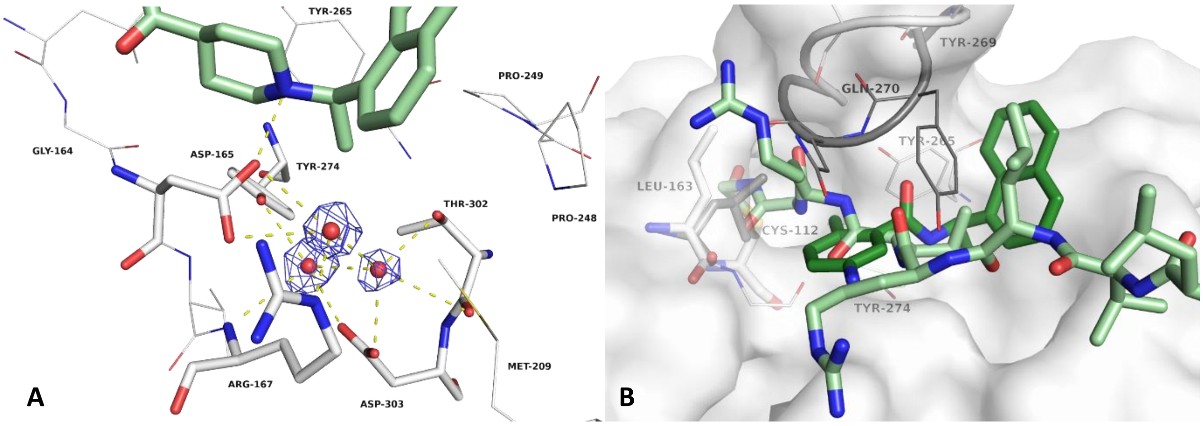

SI-Figure 4: **(A)** S4 sub-pocket close view of SARS-CoV PL^{pro}-**20** complex (PDB-ID 4OW0, resolution 2.1 Å) with highly coordinated water molecules. The ligand is depicted with green carbon atoms as sticks, the protein is shown with white carbon atoms. The 2Fo-Fc electron

density map of water molecules 1035, 1040, 1041 is contoured at 2 σ as a blue mesh. Interactions within the water network and with surrounding residues Asp165, Arg167, Tyr274, Thr302 and Asp303 are depicted as yellow dashed lines. **(B)** Crystal structure of SARS-CoV PL^{pro} (white carbon atoms) in complex with ubiquitin-aldehyde (green carbon atoms, PDB-ID 4MM3). Superposition with SARS-CoV PL^{pro}-**2b** complex reveals altered orientation of Leu163 (grey carbon atoms and dark green ligand, PDB-ID 3E9S) opening the S3 sub-pocket towards the active site. Additionally, the loop Gly267-Gly272 is not closed over the peptide as found in the SARS-CoV PL^{pro}-**2b** complex (white and grey carbon atoms and loop, respectively).

References

[1] J. Zoń, P. Miziak, N. Amrhein, R. Gancarz, *Chem. Biodivers.* 2005, *2*, 1187–1194.

[2] G. Wang, X. Yang, Y. Sun, H. Bao, X. Li, *Macromol. Chem. Phys.* 2010, *210*, 2118–2124.

[3] M. P. Hay, K. O. Hicks, K. Pchalek, H. H. Lee, A. Blaser, F. B. Pruijn, R. F. Anderson, S. S. Shinde, W. R. Wilson, W. A. Denny, *J. Med. Chem.* 2008, *51*, 6853–6865.

[4] C. Maiereanu, C. Schmitt, N. Schifano-Faux, D. Le Nouën, A. Defoin, C. Tarnus, *Bioorganic Med. Chem.* 2011, *19*, 5716–5733.

[5] Y. M. Báez-Santos, S. J. Barraza, M. W. Wilson, M. P. Agius, A. M. Mielech, N. M. Davis, S. C. Baker, S. D. Larsen, A. D. Mesecar, *J. Med. Chem.* **2014**, *57*, 2393–2412.

[6] J. Osipiuk, R. Jedrzejczak, C. Tesar, M. Endres, L. Stols, G. Babnigg, Y. Kim, K. Michalska, A. Joachimiak, *Cent. Struct. Genomics Infect. Dis.* 2020, DOI 10.2210/pdb6w9c/pdb.

[7] D. Shin, R. Mukherjee, D. Grewe, D. Bojkova, K. Baek, A. Bhattacharya, L. Schulz, M. Widera, A. R. Mehdipour, G. Tascher, P. P. Geurink, A. Wilhelm, G. J. van der Heden van Noort, H. Ovaa, S. Müller, K. P. Knobeloch, K. Rajalingam, B. A. Schulman, J. Cinatl, G. Hummer, S. Ciesek, I. Dikic, *Nature* 2020, 1–10.
